# Less is more: a methodological assessment of extraction techniques for per- and polyfluoroalkyl substances (PFAS) analysis in mammalian tissues

**DOI:** 10.1007/s00216-023-04867-5

**Published:** 2023-08-22

**Authors:** Helena Mertens, Benedikt Noll, Tanja Schwerdtle, Klaus Abraham, Bernhard H. Monien

**Affiliations:** Department of Food Safety, German Federal Institute for Risk Assessment (BfR), Max-Dohrn-Str. 8-10, 10589 Berlin, Germany

**Keywords:** Per- and polyfluoroalkyl substances, PFAS, Extraction, Tissues, UPLC-MS/MS

## Abstract

Per- and polyfluoroalkyl substances (PFAS) are persistent environmental contaminants. Studying the bioaccumulation in mammalian tissues requires a considerable effort for the PFAS extraction from complex biological matrices. The aim of the current work was to select and optimize the most efficient among common extraction strategies for eleven perfluoroalkyl acids (PFAA). Primary extractions from wild boar tissues (liver, kidney, and lung) were performed with methanol at neutral, acidic, or alkaline conditions, or with methyl-*tert*-butyl ether (MTBE) after ion-pairing with tetrabutylammonium (TBA) ions. A second purification step was chosen after comparing different solid-phase extraction (SPE) cartridges (Oasis WAX, ENVI-Carb, HybridSPE Phospholipid) and various combinations thereof or dispersive SPE with C18 and ENVI-Carb material. The best extraction efficiencies of the liquid PFAA extraction from tissue homogenates were achieved with methanol alone (recoveries from liver 86.6–114.4%). Further purification of the methanolic extracts using dispersive SPE or Oasis WAX columns decreased recoveries of most PFAA, whereas using pairs of two SPE columns connected in series proved to be more efficient albeit laborious. Highest recoveries for ten out of eleven PFAA were achieved using ENVI-Carb columns (80.3–110.6%). In summary, the simplest extraction methods using methanol and ENVI-Carb columns were also the most efficient. The technique was validated and applied in a proof of principle analysis in human tissue samples.

**Graphical Abstract:**

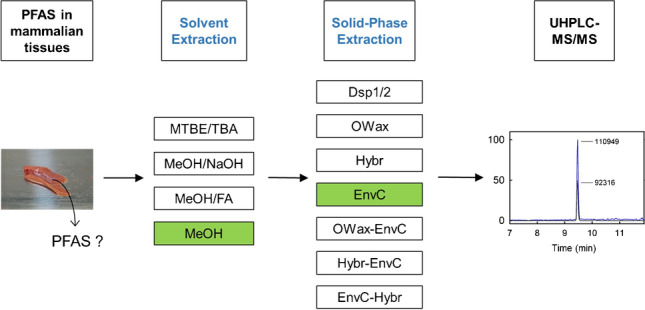

**Supplementary information:**

The online version contains supplementary material available at 10.1007/s00216-023-04867-5.

## Introduction

Per- and polyfluoroalkyl substances (PFAS) are a complex group of synthetic chemicals that, according to the definition of the Organisation for Economic Co-operation and Development (OECD), contains at least one fully fluorinated methyl or methylene carbon atom [1]. A large group among PFAS is the perfluoroalkyl acids (PFAA) comprising perfluoroalkyl carboxylic acids (PFCA) and perfluoroalkyl sulfonic acids (PFSA) composed of fluorinated carbon backbones with varying chain lengths terminated by a carboxylate or a sulfonate group, respectively. Due to their unique properties of water and oil repellency, the compounds have been used for the production of many consumer products for decades.

The increasing awareness of toxicity, persistence, and mobility accelerated the development of analytical methods for PFAA quantification in different matrices by isotope-dilution liquid chromatography coupled to tandem mass spectrometry (LC–MS/MS). The detection in, e.g., water and wastewater [2] and soils and sludge [3] as well as atmospheric particulate matter [4] highlighted that PFAA release caused worldwide environmental contamination. The PFAA analyses in animals from different ecosystems allowed monitoring persistence, long-range transport potential, bioaccumulation in the food chain, and adverse effects [5–7]. Monitoring PFAA in human plasma or serum samples allowed studying the first appearances and exposure declines due to the phase-out of single PFAA over years and decades as well as their toxicokinetics [8], which assisted risk assessment and regulation. Internal exposure to PFAA in individuals revealed four compounds to typically represent more than 90% of detectable PFAA in adults in industrialized countries, namely perfluorooctane sulfonic acid (PFOS), perfluorooctanoic acid (PFOA), perfluorohexane sulfonic acid (PFHxS), and perfluorononanoic acid (PFNA) [9].

The analyses of PFAA in tissue samples of animals and humans are of great interest because it is not clear whether and to what extent specific PFAA accumulate and exert local toxic effects. For example, it was suggested that pulmonary accumulation of perfluorobutanoic acid (PFBA) may aggravate the course of SARS-CoV-2 infections [10]. A recent analysis showing that PFBA was barely detectable in human lung tissue put this hypothesis into question [11]. Achieving a better overview of potential local accumulation requires robust and sensitive analytical methods. The main challenge for the simultaneous quantification of multiple PFAA with varying chemical properties by isotope-dilution LC–MS/MS is to find a technique for the extraction from tissues that efficiently depletes components from the complex biological matrices interfering with mass spectrometric detection of PFAA. In light of the variety of available sample preparation methods [12–14], it is difficult to choose the most efficient one assuring the highest detection sensitivities.

**Fig. 1:**
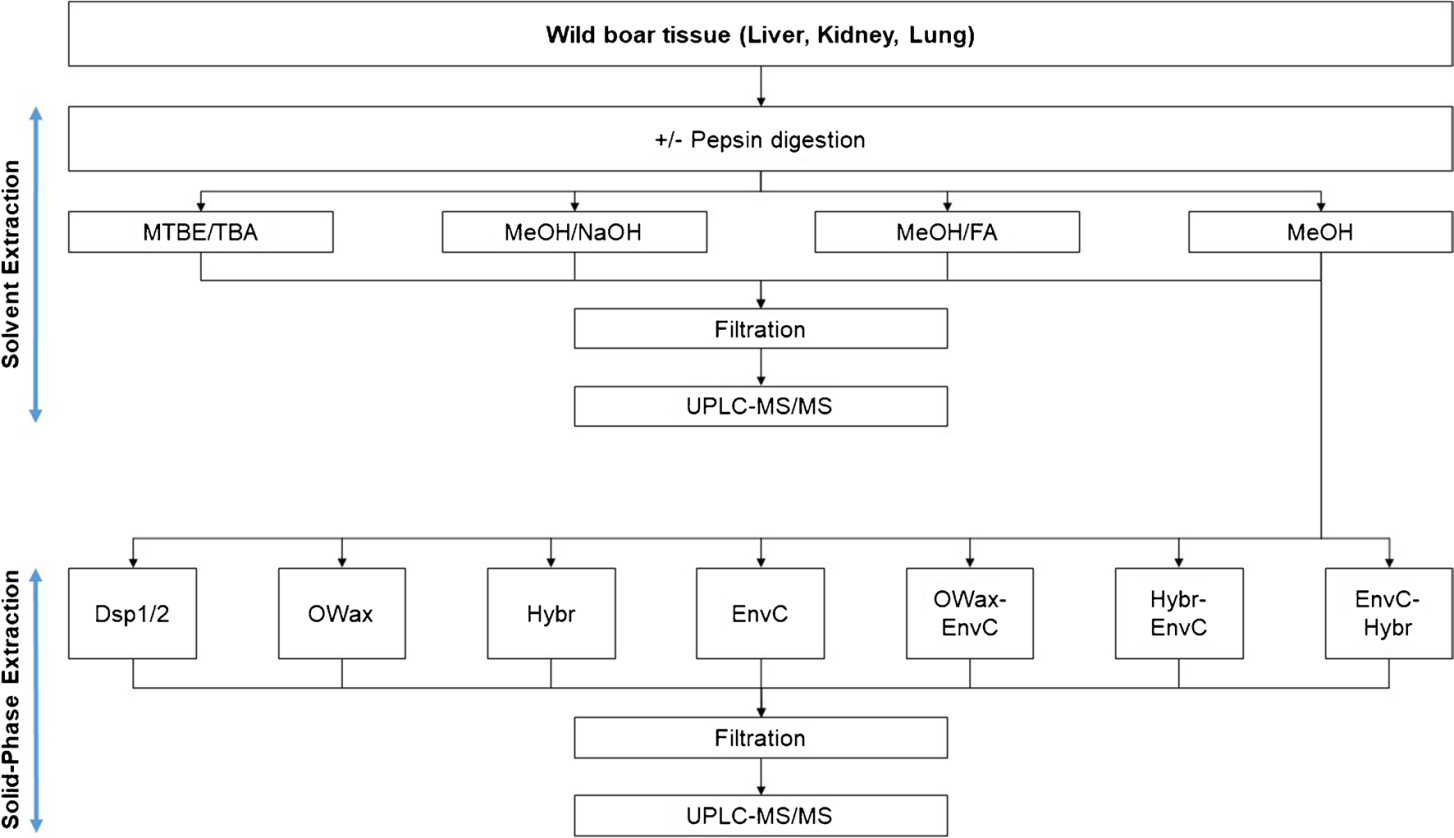
Development of PFAA extraction from homogenates of wild boar tissues. Solvent extractions were applied with methyl-*tert*-butylether and tetrabutylammonium (*MTBE/TBA*), methanol in the presence of sodium hydroxide (*MeOH/NaOH*) or formic acid (*MeOH/FA*), or methanol alone (*MeOH*). It was also tested whether the pre-incubation with pepsin supports the extraction (± *pepsin digestion*). Different SPE methods were tested for the further enrichment of PFAA using dispersive SPE (*Dsp1 and Dsp2*), the SPE columns Oasis WAX (*OWax*), HybridSPE Phospholipid (*Hybr*), ENVI-Carb (*EnvC*), or the combinations of the columns *OWax-EnvC*, *Hybr-EnvC*, or *EnvC-Hybr*

In the present work, we have combined and compared several methods. Four different ways of liquid extraction techniques were applied for the primary extraction of PFAA from tissue homogenates (Fig. 1), using methanol without additives (*MeOH*) [15–17], or in the presence of formic acid (*MeOH/FA*) [18–21] or sodium hydroxide (*MeOH/NaOH*) [21–23]. Alternatively, the homogenates were slurried with water and PFAA were extracted by methyl-*tert*-butyl ether (MTBE) after the addition of the ion-pairing tetrabutylammonium (TBA) [24, 25]. It was also tested whether the extraction is supported by a preceding incubation with pepsin for the digestion of proteins [26]. Eight SPE methods were compared for further enrichment of PFAA and matrix depletion following the primary solvent extraction (Fig. 1): two approaches of dispersive SPE with C18 and ENVI-Carb (*EnvC*) bulk material (*Dsp1* and *Dsp2*) [18, 19], three different SPE cartridges, the *EnvC* column [17], the Oasis WAX (*OWax*) column [11], and the HybridSPE phospholipid (*Hybr*) column [15], and three of the possible permutations (*OWax-EnvC*, *Hybr-EnvC*, and *EnvC-Hybr*). The objectives of this study were to evaluate the advantages and disadvantages of the strategies at hand of three parameters. First, we determined the recovery of spiked isotope-labeled standards of eleven legacy PFAA from homogenate samples of wild boar liver, kidney, and lung. The spike experiment may not be able to represent the efficacy of dissociation of protein-bound PFAA in the real sample. Therefore, we recorded also the extraction efficiencies and signal-to-noise ratios (S/N) observed for the unlabeled PFAA observed in wild boar tissues. The technique combining the best extraction method with a sensitive analysis by isotope-dilution ultra-performance liquid chromatography coupled to tandem mass spectrometry (UPLC-MS/MS) was validated. As a proof of principle, the PFAA contents of small sets of human samples from the lung (*n* = 8), colon (*n* = 5), and kidney (*n* = 3) were determined.

## Material and methods

### Human and wild boar tissue samples

Anonymized tissue samples from tumor patients collected between 2011 and 2014 in France were provided by Biopredic International (Rennes, France). The patients gave their informed consent for the collection of non-neoplastic surgical leftovers and their further use in scientific research. Following interventions, the tissue samples from the lung (*n* = 8), colon (*n* = 5), and kidney (*n* = 3) were stored at − 80 °C. Samples of the liver, kidney, and lung were collected from wild boars after driven hunts in Berlin and Brandenburg (Germany) organized by the German Institute for Federal Real Estate (BImA) [27]. Human and wild boar tissue samples (5 to 10 g) were immersed in liquid nitrogen, homogenized using a Tube Mill 100 control (IKA, Staufen, Germany), and the homogenates were stored at − 80 °C.

### Materials

Formic acid (reagent grade, ≥ 95%), 7 N ammonia in methanol, pepsin (≥ 250 units/mg solid), and tetrabutylammonium hydrogen sulfate were from Sigma-Aldrich (Darmstadt, Germany). Ammonium acetate was from Fluka (Buchs, Switzerland). Sodium bicarbonate, sodium hydroxide, and magnesium sulfate (all pro analysi grade) were purchased from Merck (Darmstadt, Germany). HPLC-grade water was prepared using a Milli-Q Integral Water Purification System from Millipore Merck (Darmstadt, Germany). Methanol (LC–MS grade), methyl-*tert*-butyl ether (MTBE; GC–MS grade), and acetonitrile (hypergrade) were from Merck. A mixture of isotope-labeled PFAA (MPFAC-24ES) was purchased from Wellington Laboratories Inc. (Guelph, Canada). It contained ^13^C_5_-PFHxA (M5PFHxA), ^13^C_4_-PFHpA (M4PFHpA), ^13^C_8_-PFOA (M8PFOA), ^13^C_9_-PFNA (M9PFNA), ^13^C_6_-PFDA (M6PFDA), ^13^C_7_-PFUdA (M7PFUdA), ^13^C_2_-PFDoA (MPFDoA), ^13^C_2_-PFTeDA (M2PFTeDA), ^13^C_3_-PFBS (M3PFBS), ^13^C_3_-PFHxS (M3PFHxS), and ^13^C_8_-PFOS (M8PFOS).

Supelclean ENVI-Carb columns (250 mg, 6 mL, Supelco), Supelclean ENVI-Carb bulk material (Supelco), and HybridSPE Phospholipid columns (30 mg, 1 mL, Supelco) were from Merck (Darmstadt, Germany). Oasis WAX columns (150 mg, 6 mL) were obtained from Waters (Eschborn, Germany) and C18 Bondesil bulk material (40 µm) was from VWR International (Darmstadt, Germany). Regenerated cellulose filters (0.2 µm, 13 mm) were acquired from WICOM (Heppenheim, Germany).

### Dilution of isotope-labeled PFAA

A stock solution of isotope-labeled PFAA (IS) containing nominal concentrations of about 10 µg/L was prepared by dilution of 250 µL MPFAC-24ES (containing isotope-labeled PFAA at 1 µg/mL) in 25 mL methanol using a volumetric flask. The resulting concentrations were re-determined with four calibration solutions (nominal concentrations 10 µg/L) prepared independently from MPFAC-24ES by stepwise gravimetric dilution in methanol. The IS solution was aliquoted for further use and stored at − 80 °C.

### Solvent extraction methods

Four techniques were compared for the extraction of isotope-labeled PFAA from wild boar tissues with or without a preceding pepsin digestion (Fig. 1).

1. Methyl-*tert*-butyl ether/tetrabutylammonium ion *(MTBE/TBA)*. Before PFAA extraction using MTBE, tissue homogenate samples (~ 0.5 g) were slurred with 4 mL of water and 20 µL IS (nominal concentrations of 10 µg/L isotope-labeled PFAA) was added. An aliquot of one-fourth of the samples (1.13 mL) was mixed with 1 mL of TBA solution (0.5 mol/L), 2 mL of sodium carbonate buffer (0.25 mol/L, pH 10), and 5 mL of MTBE. After thorough shaking (20 min), samples were centrifuged at 2500 × g (10 min). The clear organic phase was removed. The extraction was performed a second time and the extracts were combined.
2. Methanol *(MeOH)*. In the case of the methanol extraction at neutral pH, tissue homogenate samples (~ 0.5 g) were mixed with 20 µL IS and 5 mL of methanol. The samples were vortexed for 60 s, thoroughly mixed (10 min), and sonicated (20 min). After centrifugation at 2500 × g (10 min), the supernatant was removed. The extraction was repeated and the extracts were combined.
3. Methanol/formic acid *(MeOH/FA)*. The extraction was performed essentially as described for *MeOH* using two times 5 mL of methanol/formic acid (1:1). The combined extracts were neutralized by the addition of 1.5 mL 8 M sodium hydroxide and centrifuged at 2500 × g (10 min).
4. Methanol/sodium hydroxide *(MeOH/NaOH)*. The alkaline extraction followed the same procedure as described for *MeOH* using two times 5 mL of 100 mM sodium hydroxide in methanol [21]. The combined supernatants were neutralized by adding 30 µL formic acid and centrifuged at 2500 × g (10 min).

For the evaluation of the extraction efficiencies, the total extract of technique 1 or one-fourth of the extracts (technique 2: 2.63 mL; technique 3: 3.0 mL; technique 4: 2.64 mL) was concentrated in a stream of nitrogen to about 200 µL. The residuals were filled up to 1 mL with methanol and then 1 mL of 2 mM aqueous ammonium acetate was added. After centrifugation at 2500 × g (10 min, 10 °C), the supernatants were filtered through regenerated cellulose syringe filters (0.2 µm, 13 mm) and PFAA were analyzed by UPLC-MS/MS.

The influence of a preceding digestion with pepsin was tested as follows. Samples of wild boar tissue homogenates (0.5 g) were mixed with 20 µL of IS and 5 mL of a 10 g/L pepsin solution in water. After vigorous shaking (10 min), the pH was adjusted to 2.5 with formic acid, and the sample was incubated for 16 h at 37 °C. The mixture was sonicated (15 min) and pepsin was inactivated in boiling water (10 min). After neutralization with 850 µL 8 M aqueous sodium hydroxide, one-fourth of the sample was extracted with one of the methods described above.

### Solid-phase extraction (SPE) methods

The SPE methods were tested with three different extracts from homogenates of wild boar liver, kidney, and lung using methanol, which were concentrated to 200–400 µL under nitrogen.

1. Dispersive SPE (*Dsp1* and *Dsp2*). The dispersive SPE method was performed as described by Lacina et al*.* [18] (*Dsp1*) or with increased amounts of sorbents stated in brackets (*Dsp2*). The concentrated extracts were filled up with 1 mL (5 mL) acetonitrile, vortexed, sonicated, and centrifuged at 2500 × g (10 min). The supernatants were mixed with 20 mg (100 mg) of Bondesil C18, 10 mg (50 mg) of *EnvC* bulk material, and 200 mg (1 g) of magnesium sulfate. After shaking for 20 s, the mixtures were centrifuged at 2500 × g (10 min) and the supernatants were removed.
2. Oasis WAX (*OWax*). The columns were conditioned with 12 mL of 0.15% ammonia in methanol, 4 mL of methanol, and 4 mL of water. The concentrated extracts were diluted with 2.5 mL methanol and 2.5 mL 2 mM aqueous ammonium acetate, vortexed, sonicated, and centrifuged at 2500 × g (10 min). After loading the supernatants, the columns were washed with 4 mL of 25 mM aqueous ammonium acetate and 4 mL of methanol. PFAA were eluted with 8 mL of 0.15% ammonia in methanol.
3. HybridSPE Phospholipid cartridge (*Hybr*). The method was adapted from Trimmel et al. [15] with slight modifications. Concentrated extracts were diluted with 1.2 mL 100 mM ammonium acetate in methanol and 500 µL water. The samples were vortexed, sonicated, and centrifuged at 2500 × g (10 min), and the supernatants were loaded onto the *Hybr* cartridges. PFAA were eluted with 2 mL water/100 mM ammonium acetate in methanol (1:3).
4. ENVI-Carb cartridge (*EnvC*). The columns were conditioned with 4 mL 100 mM ammonium acetate in methanol and 4 mL 2 mM aqueous ammonium acetate solution. The concentrated extracts were diluted with 1 mL methanol, vortexed, and sonicated. After adding 4 mL 2 mM aqueous ammonium acetate solution, samples were vortexed and centrifuged at 2500 × g (10 min). The supernatants were loaded and the flow-through was discarded. PFAS were eluted with 2 mL of 100 mM ammonium acetate in methanol.
5. ENVI-Carb and HybridSPE Phospholipid cartridges (*EnvC*-*Hybr*). After extraction by the *EnvC* columns as described above (4), the eluates were diluted with 667 µL of water and loaded onto the *Hybr* cartridges. PFAA were eluted with 1 mL water/100 mM ammonium acetate in methanol (1:3).
6. HybridSPE Phospholipid and ENVI-Carb cartridges (*Hybr-EnvC*). The first step of the purification was performed as described (3). The eluates were applied to *EnvC* cartridges pre-conditioned with 4 mL of methanol and 4 mL of water/100 mM ammonium acetate in methanol (1:3). The elution was completed with 2 mL of 100 mM ammonium acetate in methanol.
7. Oasis WAX and ENVI-carb cartridges (*OWax-EnvC*). The columns stacked in series were conditioned with 12 mL of 0.15% ammonia in methanol, 4 mL of methanol, and 4 mL of water. The extracts were diluted with 2.5 mL methanol and 2.5 mL 2 mM aqueous ammonium acetate, vortexed, sonicated, and centrifuged at 2500 × g (10 min). The supernatant was loaded and the columns were washed with 4 mL of 25 mM aqueous ammonium acetate and 4 mL of methanol. PFAA were eluted with 8 mL of 0.15% ammonia in methanol.

After SPE, the columns were dried using a gentle vacuum. For UPLC-MS/MS analysis, the extracts were evaporated to dryness under nitrogen before being resuspended in 100 µL methanol and 100 µL of 2 mM aqueous ammonium acetate. Samples were transferred to polypropylene tubes, centrifuged at 18,000 × g (10 min, 10 °C), and the supernatants were filtered through regenerated cellulose syringe filters (0.2 µm, 13 mm).

### UPLC-MS/MS analysis

The samples were analyzed using an I-Class UPLC (Waters) connected to a QTrap6500 mass spectrometer (Sciex, Darmstadt, Germany) equipped with an electrospray ionization (ESI) source working in the negative mode. The target PFAS analytes injected in samples of 10 µL were separated by reversed-phase chromatography on a HSS T3 column (2 mm × 150 mm, 1.8 µm, Waters) using a gradient of 2 mM ammonium acetate in water/methanol (95:5, solvent A) and 2 mM ammonium acetate in methanol (solvent B). The gradient applied at a flow rate of 0.3 mL/min was: 0 min (0% B), 10 min (95% B), 11 min (95% B), 11.1 min (0% B), 14 min (0% B).

The temperatures of the sample manager and column oven were set to 8 °C and 35 °C, respectively. The mass spectrometer was operated with the following parameters: curtain gas, 20 psi; collision-activated dissociation (CAD) gas, medium; ion source temperature, 450 °C; ion spray voltage, 5500 V; ion source gas 1, 60 psi; ion source gas 2, 50 psi. PFAA and the isotope-labeled standards were detected by multiple reaction monitoring (MRM). The recorded transitions and the respective detection parameters, especially declustering potentials and collision energies, are summarized in Table S1 of the Supplementary Information. Data acquisition and processing were done with Analyst 1.7.1 software (Sciex).

### Validation

Due to the presence of background PFAA in liver tissue, the best method selected combining a primary extraction with methanol (*MeOH*) with a further purification with ENVI-Carb cartridges (*EnvC*) was validated with isotope-labeled PFAA (MPFAC-24ES). The linear ranges of mass spectrometric PFAA detection were determined using 16 concentrations prepared from MPFAC-24ES between 0.00025 and 25 µg/L, diluted in 2 mM aqueous ammonium acetate/methanol (1:1). In order to mimic experimental conditions, the solutions were prepared in triplicates in the presence of methanolic extracts from 0.5 g liver tissue, one-fourth of which was further purified by *EnvC* columns as described above. The isotope-labeled PFAA were determined by UPLC-MS/MS MRM. The lower limits of detection (LOD) and quantification (LOQ) were defined by S/N ≥ 3 and S/N ≥ 10, respectively. The intraday precision was determined after spiking samples of 0.5 g liver homogenate with five different amounts of MPFAC-24ES (nominal values 0.0032, 0.016, 0.08, 0.4, and 2.0 µg/kg, *n* = 6). The samples were extracted with the selected method and analyzed by UPLC-MS/MS. The interday precision from the analyses was determined with the same sample sets extracted in different weeks (*n* = 6).

### Statistics

Statistical analyses were conducted with SigmaPlot version 14.0 (Systat Software, Inc., Erkrath, Germany). PFAA contents were reported as mean values (± standard deviations) of three to six independent samples.

## Results

### Liquid extraction of tissue PFAA

**Fig. 2:**
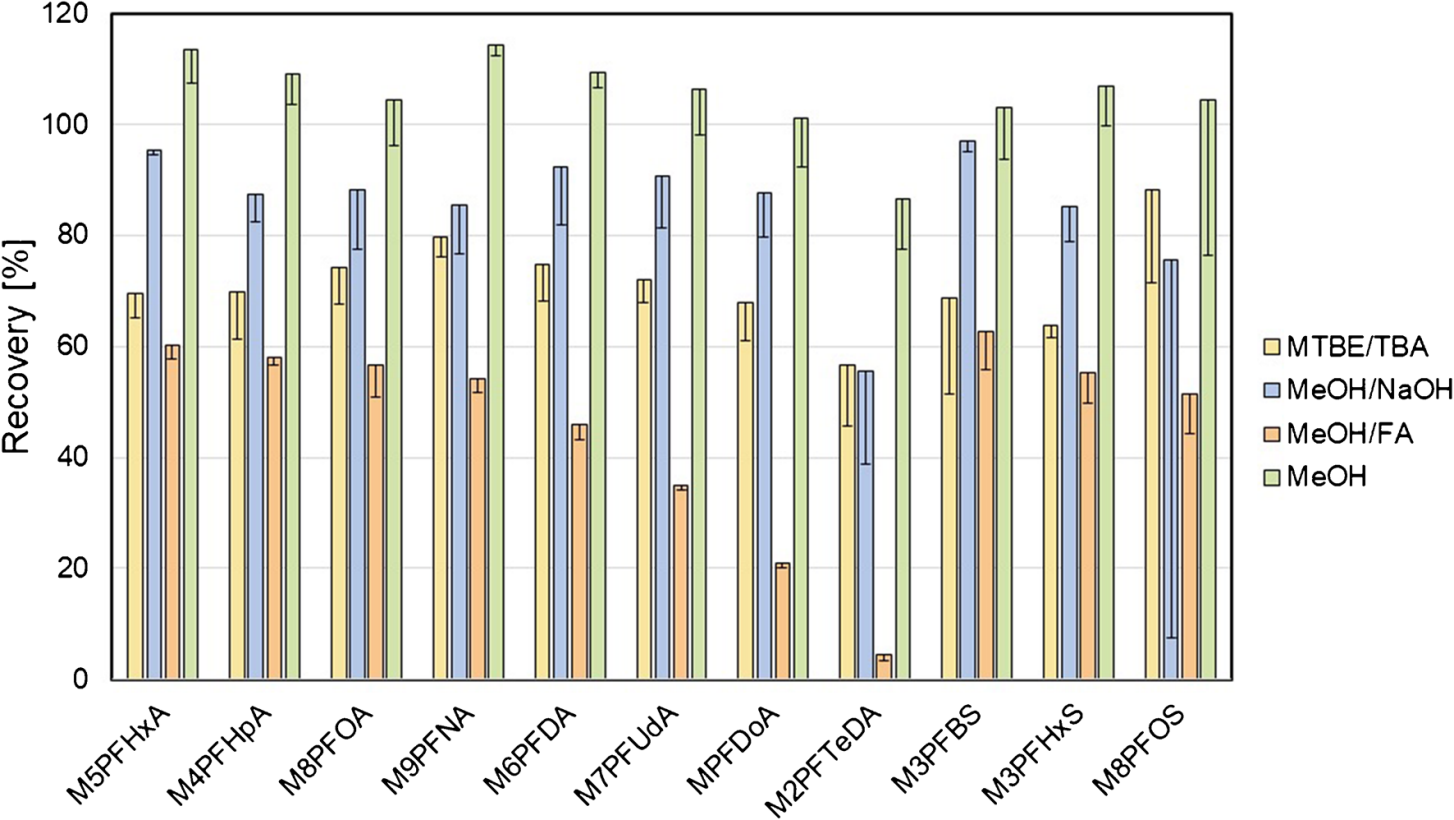
Absolute recoveries (%) after fortification of liver homogenates with isotope-labeled PFAA (~ 190 ng/g) and extraction with MTBE after addition of TBA (yellow), sodium hydroxide in methanol (blue), formic acid in methanol (orange), or methanol alone (green). The bars and error bars show means and standard deviations of three samples. The recovery data from homogenates of kidney and lung are summarized in Fig. S1 of the Supplementary Information

The extraction efficiencies of the four methods assessed by the absolute recovery of 11 isotope-labeled PFAA standards from wild boar homogenates are summarized in Fig. 2. Regardless of the method, the extraction recovery tended to decrease with the chain length of the PFAA. This was particularly obvious for the extraction with acidified methanol (*MeOH/FA*) but also if MTBE was used. The overall recoveries were higher with methanol without additives (*MeOH*: 86.6–114.4%) compared to those observed under alkaline (*MeOH/NaOH*: 55.7–96.9%) or acidic conditions (*MeOH/FA*: 4.4–62.6%). In addition, the recoveries of PFAA extracted as TBA ion pairs with MTBE were smaller (*MTBE/TBA*: 56.7–88.3%) compared to those achieved with methanol. The recovery trends were basically the same if isotope-labeled PFAA were extracted from homogenates of kidney or lung tissue (Fig. S1 of the Supplementary Information).

**Fig. 3:**
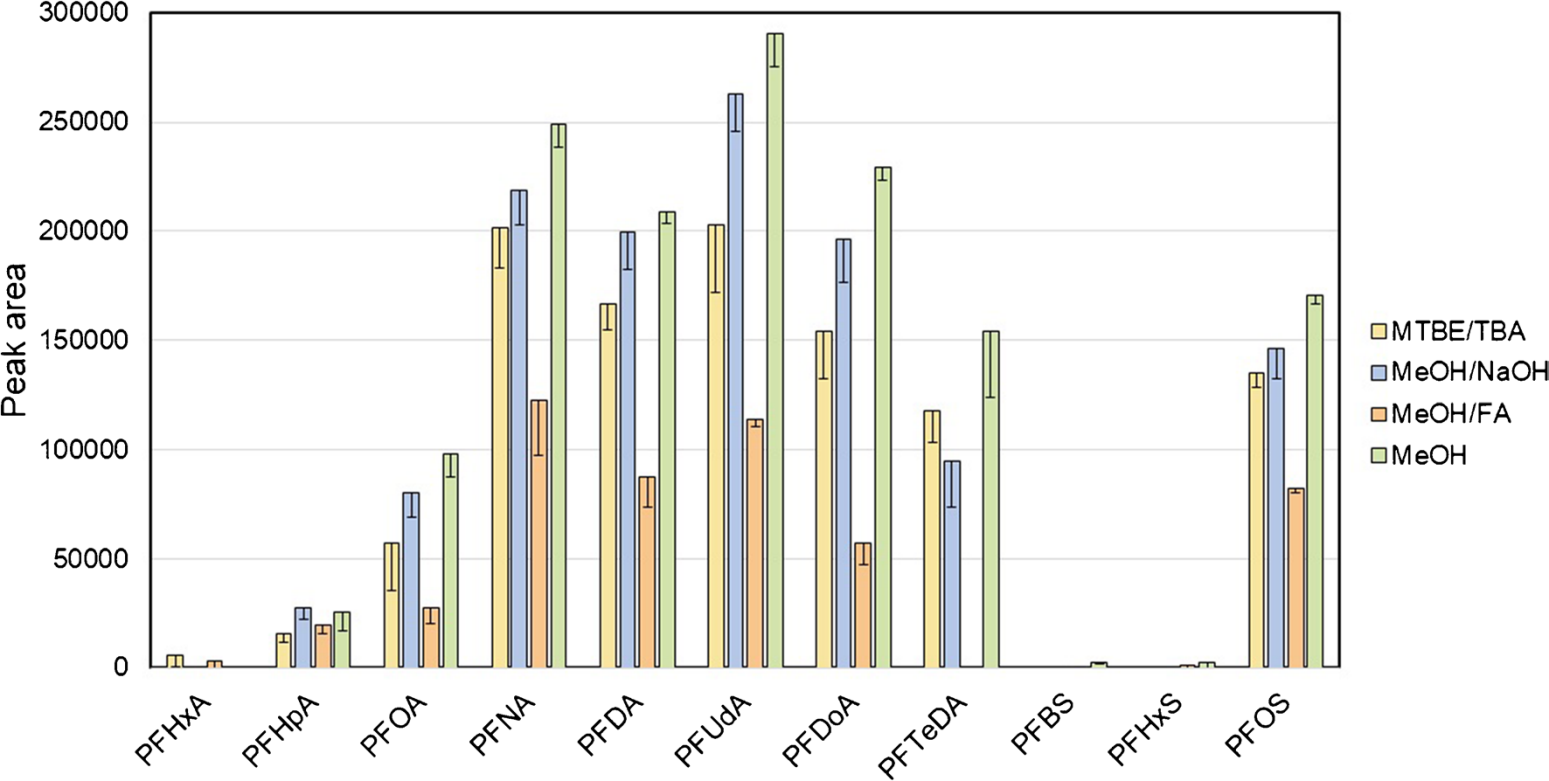
Signal intensities reflect recoveries of PFAA extractions from liver PFAA using MTBE after addition of TBA (yellow), sodium hydroxide in methanol (blue), formic acid in methanol (orange), or methanol alone (green). The bars and error bars show means and standard deviations of three samples. The recovery data from homogenates of kidney and lung are summarized in Fig. S2 of the Supplementary Information

In addition to the extraction of fortified isotope-labeled standards, we determined the extraction efficiencies for the inherent PFAA in wild boar liver homogenates (Fig. 3). Evaluation of the peak intensities confirmed the results from extraction of isotope-labeled PFAA. The extraction with methanol alone led to the highest PFAA signal intensities, which were comparatively low after the extraction of PFAA-TBA ion pairs with MTBE. Similar PFAA profiles were obtained after extraction from the kidney and lung (Fig. S2 of the Supplementary Information). It is of note that the digestion of the tissue samples using pepsin [26], an aspartic protease for the hydrolysis of proteins into peptides and amino acids, preceding the extraction of PFAA with methanol did not have a beneficial effect on the extraction (Fig. S3 of the Supplementary Information).

Besides the amount of PFAA (reflected by the signal intensities), the quantity of undesirable matrix, which may interfere with further sample processing and UPLC-MS/MS analysis, must be taken into account for the evaluation of the extraction quality. The visual impressions of the primary extracts indicated that the use of *MTBE/TBA* or *MeOH* without additives resulted in the cleanest extracts. This was confirmed by inspection of the chromatograms reflecting the presence of higher or lower matrix amounts in the samples. To substantiate this observation, we summarized the S/N for the signals of individual PFAA after extraction from the liver, kidney, and lung with the four methods in three Tables S2, S3, and S4 in the Supplementary Information. For this purpose, the noise was defined by a one-minute background signal interval before the retention time of a PFAA analyte signal. The number of detectable PFAA in the tissue homogenates (Table S2–S4) and the S/N of the peaks as auxiliary parameter supported the impression that the extractions using *MTBE/TBA* or *MeOH* were cleaner compared to those using *MeOH/NaOH* or *MeOH/FA*.

### PFAA enrichment and matrix depletion of primary tissue extracts

**Fig. 4:**
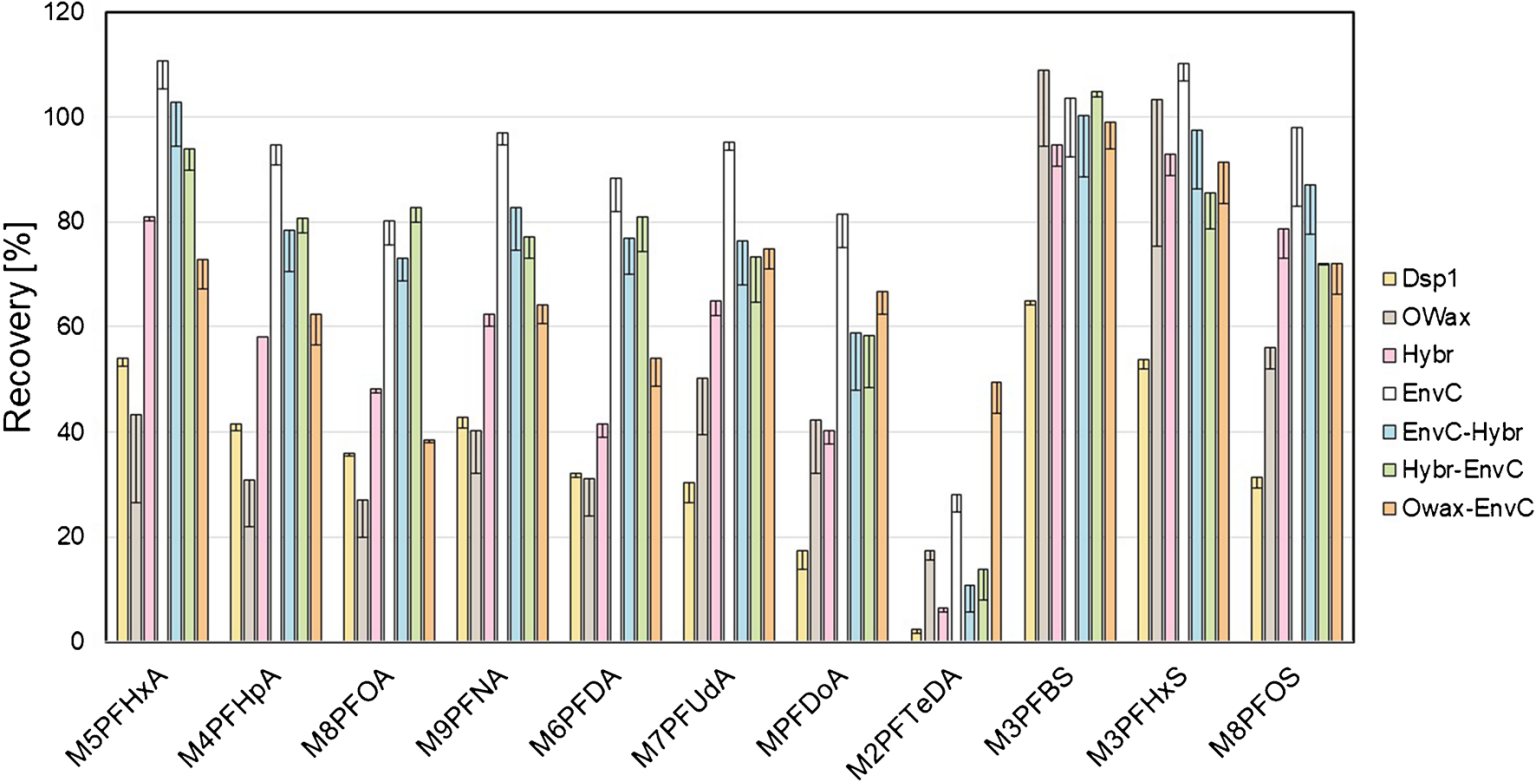
Absolute recoveries of isotope-labeled PFAA (%) from fortified wild boar liver homogenate after methanolic extraction and application of different SPE methods using dispersive SPE *Dsp1* (yellow), the SPE columns *OWax* (grey), *Hybr* (pink), or *EnvC* (white), or the combinations of the columns *EnvC-Hybr* (blue), *Hybr-EnvC* (green), or *OWax-EnvC* (orange). Results from *Dsp2* were very similar to those of *Dsp1* and omitted for clarity. The bars and error bars show means and standard deviations of three samples. The absolute recoveries of extractions from kidney and lung homogenates are summarized in Fig. S4 of the Supplementary Information

The absolute recoveries of eight SPE methods were determined using methanolic extracts of PFAA from the liver, kidney, and lung homogenates. Figure 4 summarizes the results after extraction of the fortified isotope-labeled standards from the liver; the recoveries from the kidney and lung are shown in Fig. S4 in the Supplementary Information. The results of M2PFTeDA extractions were excluded from the following comparison. Its hydrophobicity diminished the effectiveness of most SPE techniques. These require usually at least partially aqueous solutions for sample applications, which is the probable reason for the general low recoveries of the least soluble M2PFTeDA and further discussed below.

The dispersive SPE did not lead to satisfactory PFAA recoveries from the liver of at most 65% (*Dsp1*) and 62% (*Dsp2*) observed for M3PFBS. The sample enrichment with *OWax* alone showed good recoveries for M3PFBS (108.9%) and M3PFHxS (103.4%) but mediocre results for isotope-labeled PFCA (27.1–50.2%). In comparison, all methods with two SPE columns connected in series were more effective with recoveries between 58.8 and 103.0% (*EnvC-Hybr*), 58.4 and 104.9% (*Hybr-EnvC*), and 38.5 and 98.9% (*OWax-EnvC*). The results of *OWax-EnvC* were slightly superior for the extraction of long-chain PFAA (e.g., M2PFTeDA), while the combinations of *Hybr* and *EnvC* columns led to better extractions of shorter PFAA (M5PFHxA to M6PFDA). Remarkably, the analyte enrichment/matrix depletion using these columns alone—*Hybr* or *EnvC*—was also very effective with recoveries between 40.4 and 94.6% or 80.3 and 110.6%, respectively. Similar relative recoveries were observed for the extractions from other tissues (Fig. S4). The extraction with *EnvC* alone (78.0 to 107.0%) worked best with kidney homogenates. In contrast, all column-based SPE techniques yielded comparative results in the case of lung tissue.

**Fig. 5:**
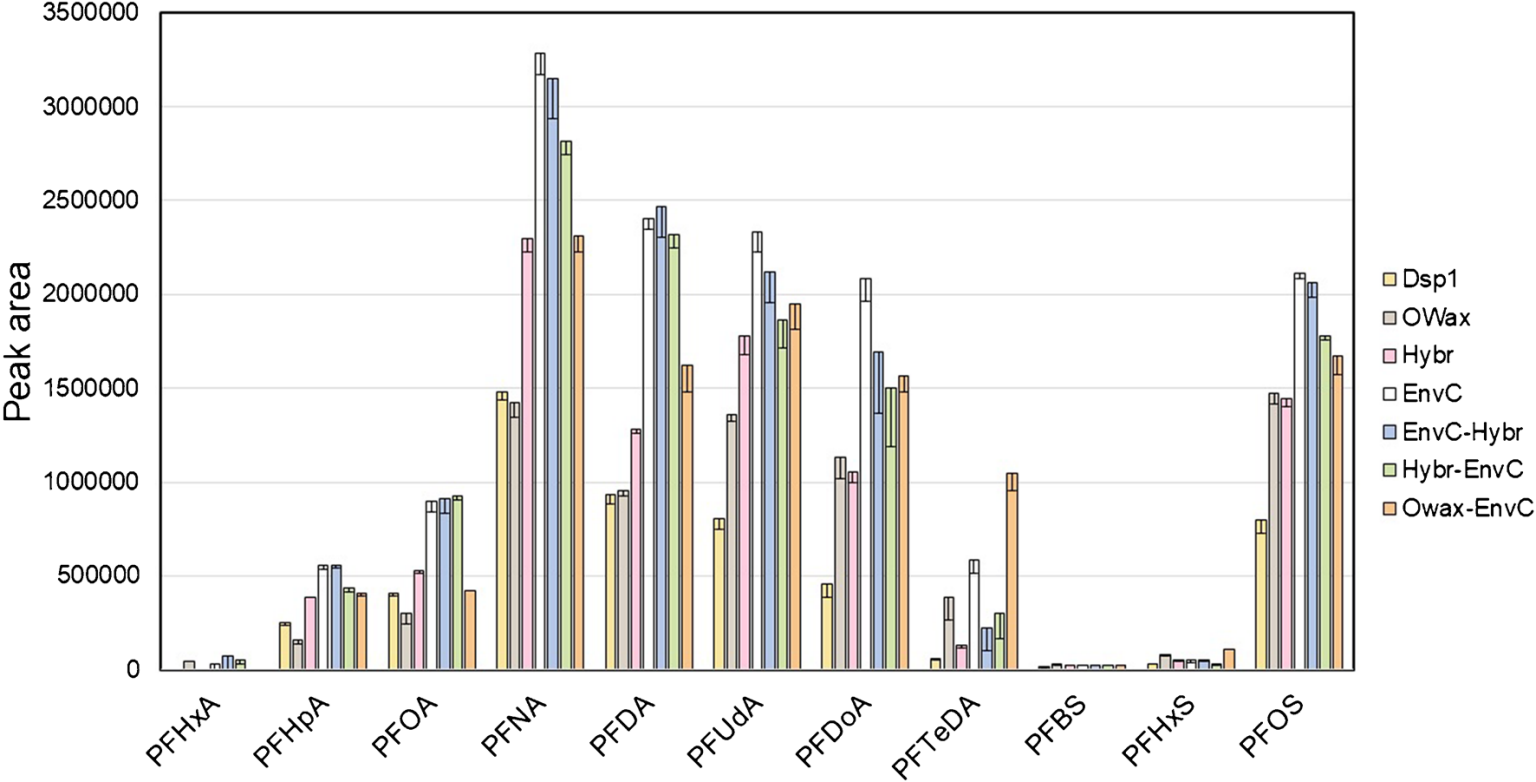
Signal intensities reflect recoveries of PFAA from wild boar liver homogenate after methanolic extraction and application of different SPE methods using dispersive SPE *Dsp1* (yellow), the SPE columns *OWax* (grey), *Hybr* (pink), or *EnvC* (white), or the combinations of the columns *EnvC-Hybr* (blue), *Hybr-EnvC* (green), or *OWax-EnvC* (orange). Results from *Dsp2* were very similar to those of *Dsp1* and omitted for clarity. The bars and error bars show means and standard deviations of three samples. The absolute recoveries of PFAA extractions from kidney and lung homogenates are summarized in Fig. S5 of the Supplementary Information

The second parameter set for the evaluation of the extraction efficiency was the collection of peak areas observed for the inherent PFAA in the tissue samples (Fig. 5). Similar to the extraction efficiency observed for the isotope-labeled PFAA, the dispersive SPE methods *Dsp1* and *Dsp2* showed the lowest signal intensities after purification of liver extracts. Using the *Hybr* or the *OWax* columns alone yielded slightly better results. Of the three serial SPE methods, the combinations *Hybr-EnvC* and *EnvC-Hybr* showed roughly equivalent and *OWax-EnvC* in comparison somewhat lower extraction efficiencies. Using the *EnvC* alone led to the highest peak areas for most of the PFAA in this study (Fig. 5). The relative efficacies of PFAA extractions from kidney and lung homogenates were comparable (Fig. S5).

We also considered the relative S/N of individual PFAA signals after SPE from methanolic extracts of wild boar liver as a parameter including the relative matrix depletion (Table S5). The overview suggested an order of efficacy correlated inversely with the number of non-quantifiable PFAA: *Dsp1*, *Dsp2* (4) < *Hybr*, *OWax-EnvC* (3) < *OWax* (2) < *EnvC*, *EnvC-Hybr*, *Hybr-EnvC* (1). The order of performance became even clearer in the PFAA analyses after extraction from the kidney and lung (Tables S6 and S7 in the Supplementary Information). When extracted from lung tissue, *EnvC* was the only method with S/N > 10 for the signals of all examined PFAA.

### Validation

The selected method (primary extraction with methanol and analyte enrichment/matrix depletion with *EnvC* columns) was validated using the isotope-labeled PFAA included in the standard solution MPFAC-24ES, because their common presence in liver tissue hinders the validation with unlabeled PFAA. The linearity of the detection range and the LOD and LOQ values were determined with dilution series of the isotope-labeled PFAA prepared in the presence of a matrix background resulting from the workup of a conventional sample (Fig. S6 of the Supplementary Information). The analyte concentration ranges with a linear response (between three and five orders of magnitude) and the LOQ values (between 0.0016 and 0.04 µg/kg) are summarized in Table 1. The low LOQ values can be attributed to the almost background-free MRM transitions, in part resulting from the efficient sample preparation. The decreased sensitivity observed for M5PFHxA is due to the interfering background.

**Table 1:** Validation parameters for the analyses of PFAA in wild boar liver after extraction with methanol and further enrichment using *EnvC* columns^a^

Analyte	Recovery^b^	Linear range^c^	LOQ^d^	
	%	µg/L	µg/L	µg/kg
M5PFHxA	110.6 ± 5.2	0.005–25	0.025	0.04
M4PFHpA	94.6 ± 3.8	0.0005–10	0.0025	0.004
M8PFOA	80.3 ± 4.6	0.0005–25	0.0025	0.004
M9PFNA	97.1 ± 2.4	0.0005–10	0.0025	0.004
M6PFDA	88.4 ± 6.4	0.001–25	0.005	0.008
M7PFUdA	95.1 ± 1.4	0.00025–25	0.001	0.0016
MPFDoA	81.4 ± 6.2	0.00025–25	0.001	0.0016
M2PFTeDA	28.0 ± 3.3	0.001–10	0.0025	0.004
M3PFBS	103.6 ± 11.3	0.0047–25	0.093	0.015
M3PFHxS	110.2 ± 3.4	0.0047–25	0.024	0.038
M8PFOS	98.1 ± 15.0	0.0048–25	0.024	0.038

^a^Due to the occurrence of PFAA in wild boar liver homogenates, the validation parameters were determined using the respective isotope-labeled compounds

^b^Recovery of the sample preparation; mean values and SD of three samples

^c^The lower values of the linear ranges mark the respective LODs

^d^The LOQ (µg/kg) was calculated relative to the standard procedure with 125 mg of liver tissue used for the enrichment with *EnvC* columns and a final sample volume of 200 µL

Inter- and intraday precision for all isotope-labeled PFAA are summarized in Table 2. The average coefficient of variation (CV) of the intraday precision over all PFAA tested close to their LOQ was 10.9 ± 6.1%. At concentrations 5 times higher and 25 times higher, the average CVs were 6.5 ± 2.9% and 4.4 ± 2.2% respectively. The average CV of the interday precision over all PFAA tested close to the LOQs was 14.2 ± 6.7%, and at concentrations 5 times higher and 25 times higher, the average CVs were 10.0 ± 2.6% and 5.3 ± 1.5% respectively. Most of the individual values meet the criteria for the inter- and intraday precision of biomarker analyses by chromatographic assays (not to exceed ± 15%, except ± 20% at the LOQ) stated by the Food and Drug Administration [28].

**Table 2:** Intra- and interday precision (CV in %) of six independent analyses at five different concentration levels of the isotope-labeled PFAA. The lowest concentrations (bold numbers) were in the range of 0.8 to 2.1 times the LOQ of the respective PFAA^a^

	Intraday precision (%)					Interday precision (%)				
	3.2 ng/kg	16 ng/kg	80 ng/kg	400 ng/kg	2 µg/kg	3.2 ng/kg	16 ng/kg	80 ng/kg	400 ng/kg	2 µg/kg
M5PFHxA	n.q.^b^	n.q	**3.7**	6.8	8.5	n.q	n.q	**10.1**	6.3	6.8
M4PFHpA	**16.4**	9.0	5.6	6.4	5.8	**22.4**	9.5	3.4	3.5	3.5
M8PFOA	**22.7**	9.6	2.4	8.7	3.6	**21.1**	14.1	6.5	6.9	3.0
M9PFNA	**5.6**	6.1	4.9	5.7	3.9	**3.7**	11.6	5.2	5.6	2.6
M6PFDA	n.q	**4.1**	8.9	3.4	6.3	n.q	**8.6**	9.5	6.9	4.9
M7PFUdA	**10.9**	2.8	3.3	6.9	6.1	**19.6**	15.1	4.1	5.3	6.8
MPFDoA	**14.5**	2.7	5.4	6.0	5.4	**14.7**	7.6	3.4	7.0	8.4
M2PFTeDA	**8.4**	2.6	2.3	4.4	5.3	**8.8**	8.8	5.3	6.7	5.3
M3PFBS	n.q	n.q	**6.5**	7.7	5.5	n.q	n.q	**8.4**	9.4	7.8
M3PFHxS	n.q	n.q	**7.7**	5.7	1.9	n.q	n.q	**23.2**	8.8	4.5
M8PFOS	n.q	n.q	**17.5**	10.4	3.4	n.q	n.q	**16.0**	9.3	3.8

^a^Due to the PFAA background in the liver homogenate, the precision values were determined using the isotope-labeled compounds

^b^n.q. (not quantified), signals were below the LOQ of the respective PFAA

### PFAA in human tissue samples

The method was applied to the quantification of PFAA in human tissue samples. PFOA, PFNA, PFDA, PFUdA, PFHxS, and PFOS were comfortably quantifiable in all samples analyzed, lung (*n* = 8), kidney (*n* = 5), and colon (*n* = 3). Exemplary chromatography data for the separation and the mass spectrometric detection of the seven PFAA in one sample (*lung 1*) are summarized in Fig. S7 of the Supplementary Information. The quantification results are summarized in Table 3. PFDoA was mostly and PFHpA and PFTeDA were occasionally quantifiable, whereas signals of PFHxA and PFBS were < LOQ in all samples analyzed.

**Table 3:** PFAS concentrations (µg/kg) determined in tissue samples of human lung (*n* = 8), colon (*n* = 5), and kidney (*n* = 3)^a^

	PFHpA	PFOA	PFNA	PFDA	PFUdA	PFDoA	PFTeDA	PFHxS	PFOS
Lung 1	n.q.^b^	0.69	0.22	0.13	0.14	0.045	n.q	0.63	3.89
Lung 2	n.q	1.12	0.29	0.13	0.18	0.040	n.q	0.54	4.78
Lung 3	n.q	0.99	0.52	0.43	0.29	0.075	n.q	0.47	3.35
Lung 4	0.04	1.18	0.41	0.14	0.16	0.067	0.06	0.46	2.79
Lung 5	0.05	0.78	0.16	0.08	0.06	0.022	n.q	0.42	2.87
Lung 6	n.q	0.97	0.41	0.20	0.16	0.039	n.q	0.46	4.08
Lung 7	0.04	1.04	0.32	0.12	0.08	0.024	n.q	0.31	3.12
Lung 8	n.q	0.53	0.29	0.15	0.11	0.035	n.q	0.59	4.62
Colon 1	0.04	1.14	0.25	0.11	0.10	0.025	n.q	0.34	3.35
Colon 2	n.q	0.43	0.14	0.07	0.05	0.016	n.q	0.31	1.50
Colon 3	n.q	0.48	0.20	0.10	0.11	0.031	0.02	0.41	2.59
Colon 4	n.q	0.39	0.09	0.04	0.02	n.q	n.q	0.18	0.56
Colon 5	n.q	0.19	0.07	0.04	0.01	n.q	n.q	0.26	0.70
Kidney 1	n.q	3.80	1.60	0.78	0.30	0.114	0.06	8.12	10.49
Kidney 2	0.10	1.13	0.27	0.12	0.24	0.057	0.03	2.39	3.16
Kidney 3	n.q	0.52	0.28	0.16	0.12	0.035	0.02	2.51	4.38

^a^Signals of PFHxA and PFBS were below the LOQ in all samples analyzed

^b^n.q. (not quantified), signals were below the LOQ (S/N = 10)

## Discussion

A variety of methods was devised for the extraction of PFAA, all of which exploit the relative hydrophobicity exceeding that of most, more water-soluble substances in the matrices. Consequently, extractions with organic solvents and/or reversed-phase columns are common techniques [12–14]. The material choices and resulting efficiencies depend on the matrix composition. Enrichment from dense matrices (e.g., tissue samples, food), which contain substance classes with chemical similarity to PFAA, requires greater efforts compared to the extraction from less complex matrices (e.g., drinking water, serum). Extracting a variety of PFAA from tissue samples is a particular challenge. First, the target PFAA cover a wide range of more hydrophilic (e.g., PFBS) to more hydrophobic substances (e.g., PFTeDA), which indicates that the best extraction method may probably not be suitable for all targeted PFAA in the same manner. Second, the protein binding of PFAA [3, 29, 30] and the co-extraction of peptides and (phospho-)lipids from biological samples in high amounts [15, 18, 31] are recognized challenges.

The goal of the current study was to find an optimal extraction and clean-up procedure for the target PFAA from tissue samples of mammals. With this focus, other parameters with a major influence on the sensitivity and specificity of final UPLC-MS/MS analyses were kept constant, e.g., the amount of tissue used for the solvent extraction (0.5 g) and the sample amount finally injected (10 µL). Also, the volumes of solvents used for the primary extractions (~ 10 mL) and the bed weights of the chosen SPE columns were not varied from typical setups in previous studies [11, 16, 23, 30, 32–34].

The most widely applied solvents used for the primary PFAA extraction from tissue homogenates are methanol or acetonitrile with or without pH adjustment. Acidic or alkaline conditions are believed to weaken the ionic interactions between PFAA and positively charged amino groups of amino acid side chains in proteins. To our surprise, the best results were achieved with methanol alone, whereas the presence of formic acid [18–21] or sodium hydroxide [21–23] led to problems with sample handling without improving the PFAA extraction. For example, the neutralization of the acidic extracts of wild boar tissues led to the appearance of transparent gel-like precipitates that were resistant to centrifugation and hindered the subsequent filtration. The pH adjustment of alkaline extracts entailed the formation of a solid precipitate (scavenging some of the dissolved PFAA) and the requirement of an additional centrifugation step. In contrast, neutral methanolic extracts were relatively clean as judged by the visual impression of the samples and S/N of the signals of inherent PFAA (Tables S2–S4). They contained the highest levels of isotope-labeled PFAA, showed the highest peak areas for unlabeled PFAA, and guaranteed an unhindered proceeding with further workup steps (filtration and concentration). In addition, it was the most straightforward extraction method in our study. Results from two previous reports pointed in the same direction. So et al*.* [29] evaluated the effect of alkaline extraction of PFAA from oysters and mussels with different concentrations of potassium hydroxide in water (0.01, 0.1, 0.3, 0.5, 1, and 2 N) or methanol (0.01, 0.1, and 0.3 N). The lowest concentrations of potassium hydroxide yielded the highest extraction efficiencies for the PFAS tested in the range of 100%. Unfortunately, the authors did not test the extraction in the absence of potassium hydroxide [29]. A more recent study found that, among six different solvent mixtures tested for the extraction of PFAA from plant matrices, including methanol or acetonitrile with 0.1% formic acid or methanol with 400 mM ammonium acetate, methanol alone proved to be the most efficient solvent [35].

A widely used alternative to the mentioned approaches is the conversion of PFAA to even more lipophilic ion pairs with TBA and the subsequent extraction with MTBE [24, 25, 36]. It has been described as a relatively laborious method suffering from the co-extraction of lipids and other lipophilic matrix components [18]. In the current study, the efficacy of PFAA extraction with MTBE from wild boar tissues was satisfactory. In addition, the S/N (Tables S2–S4) indicated that the extracts contained less matrix compounds compared to those obtained with *MeOH/FA* or *MeOH/NaOH*. In the case of tissue samples, the MTBE extraction may be considered a convenient one-step strategy if, for example, sample numbers are very high and the sensitivity of detection is of secondary importance.

To achieve high sensitivities of quantification by UPLC-MS/MS (and to keep the chromatography columns from wear and premature failure), the application of an SPE step following the primary solvent extraction is advised. Four SPE principles were tested in this study, *ea sunt* mixed-mode ion exchange columns (*OWax*) exploiting the amphiphilic characteristics for PFAA enrichment [11, 16, 32, 37], and graphite materials (*EnvC*), which bind PFAA by hydrophobic and van der Waals interactions basically as a reversed-phase matrix [17, 38]. Bulk C18 material was used to clean PFAA primary extracts from more hydrophobic substances [18], and the zirconia-coated silica material (*Hybr*) retains phospholipids based on a selective Lewis acid–base interaction between the zirconium ions and the phosphate groups remaining non-selective towards other acidic compounds [15]. We tested the SPE columns using 25% of the primary methanolic extracts, which is sufficient to detect the trace levels of target PFAA without overloading the chosen SPE columns with matrix content. Considering all the data (recoveries of isotope-labeled PFAA (Fig. 4 and S4), peak intensities of unlabeled PFAA (Fig. 5 and S5), and S/N of the signals (Tables S5–S7), it can be concluded that the *EnvC* column provides the best results for the concentration of the analytes and the depletion of the matrix from the primary methanolic extracts. Reviewing the procedures published previously, this is an unexpected result. The materials most commonly used for the purification of primary PFAA solvent extracts from tissues were mixed-mode anion exchangers such as *OWax* or Chromabond SB(SAX) columns [11, 12, 16, 22, 32, 34, 37], which appear to be an optimal solution considering the combination of ionic and hydrophobic interactions between PFAA and the solid-phase material. However, some studies have shown that the use of the *EnvC* can significantly improve the performance of the *OWax*. Sadia et al. [21] (PFAS extraction from food matrices including chicken meat and beef) and Zabaleta et al*.* [30] (extraction from different marine organisms) concluded that the combination *OWax-EnvC* was superior to the extraction by *OWax* columns alone [21, 30]. A similar improvement using the *Hybr* columns was observed here when extended with the *EnvC*.

Recent studies indicated before that the performance of the *EnvC* alone could not be improved by any of the other columns used. For example, Nassazzi et al*.* [35] observed that the combination of *OWax* and *EnvC* columns for the purification of a primary methanolic PFAA extract of plant material did not improve the recovery compared to the application of the *EnvC* column alone (a slight reduction of the matrix effect was overcompensated by a decrease of absolute PFAA recovery). The strength of the *EnvC* material for the extraction of PFAA can be described as follows. The manufacturer recommends the application as a reversed-phase cartridge in order to isolate amphiphilic analytes from more polar compounds [38]. Accordingly, we added sample extracts in mixtures of methanol and 2 mM aqueous ammonium acetate (1:4). PFAA were retained by the material allowing the separation of more hydrophilic compounds by column washing. The second binding principle is based on π-π interactions with the graphite surface and leads to the specific retention of matrix compounds with conjugated double bonds or aromatic character [33]. This retention is not disrupted, when PFAA are eluted with methanol. It is important to note that all studies consulted for this work used the *EnvC* columns directly after primary extraction, applying PFAA dissolved in methanol or acetonitrile. In these cases, but also when *EnvC* was used as non-retentive absorption material added directly to the primary extracts [18, 19, 39, 40], the material works as an adsorbent for molecules that are either planar (π-π interactions) or even more hydrophobic compared to PFAS, which usually has a decolorizing effect [17, 21, 41–43]. However, this does not exploit the full potential of the material and is in disagreement with the recommended use of the company [38]. The application of the *EnvC* as a reversed-phase column for the PFAA purification from primary extracts of wild boar tissues as described in this work ensured an efficiency of matrix depletion that was superior to the other columns used alone (*OWax* and *Hybr*), but also to the dispersive SPE or to combinations of two of the SPE cartridges.

A slight limitation of the method was the association between increasing chain lengths and low recoveries. This culminated in the poor extraction observed for M2PFTeDA and may be due to poor solubility in partially aqueous solutions. For example, it was necessary to dissolve the final extraction samples in methanol and 2 mM aqueous ammonium acetate (1:1) for UPLC-MS/MS analysis. This solvent ratio was chosen as a compromise between two conflicting requirements. For the separation by UPLC, dissolution of PFAA with the starting eluent would be advisable (2 mM ammonium acetate in water/methanol (95:5)). This hinders the dissolution and promotes the sorption in laboratory containers depending on the ratio of organic to aqueous solvent and the hydrophobicity of the PFAA [44]. To increase the solubility, it would be better to apply pure methanol, which, however, impairs the chromatographic separation of short-chain PFAA. For these reasons, the aforementioned solvent mixture was used when the PFAA were dissolved (and for the application of the *EnvC* columns), leading to satisfactory extraction efficacies for the second-longest PFAA (MPFDoA) and all shorter compounds, but not for M2PFTeDA.

As a proof for the applicability of the method, we analyzed the PFAA in a set of human tissue samples from the lung, colon, and kidney (Table 3). In the same set of human lung samples, the PFBA levels were already determined in a previous study using high-resolution mass spectrometry, as required for a specific detection of this compound [11]. In this study, colon samples were excluded, because the extracts using alkaline methanol and Oasis WAX columns were not sufficiently clean for the injection into the UPLC-MS. With the current technique, even the colon samples conceived earlier as problematic were extracted easily without appearances of precipitates or turbidities. It is of further note, that in agreement with the previous observation of low PFBA levels in lung and kidney samples, the current data do not support the suggestion of Perez et al. [45] that human tissues, especially lung, may accumulate PFAA.

## Conclusion

The techniques of PFAA extractions prior to LC–MS/MS analysis developed in the past decades were not especially optimized for mammalian tissues because extraction efficiencies and mass spectrometric sensitivities were sufficient for the detection of most compounds. However, future research requires extraction tools allowing to reach lower limits for the mass spectrometric quantification, because the tightening regulation of legacy PFAA in the USA and the European Union will lead to decreasing PFAA amounts in environmental matrices (e.g., plants, soil, and animals) and in humans. At the same time, the global awareness of toxicological relevance increases.

The thorough comparison of various strategies of a two-step sample preparation for the analysis of PFAA in mammalian tissue samples presented here showed that the simplest methods worked best. A primary PFAA extraction from tissue homogenates with methanol alone is the most effortless, less time-consuming, and the cheapest of all methods tested. The alkaline or acidic conditions previously often used to ensure the release of bound PFAA from proteins and lipids were detrimental to the working routine and the recoveries. The analyte enrichment/matrix depletion by SPE using an *EnvC* column did not only yield the highest PFAA recoveries, but it is also the simplest column-based method of all approaches tested. The future replacement of the *OWax* columns (150 mg) previously used in our laboratory [11] with *EnvC* columns (250 mg) reduces the cost per sample by about 20%. A limitation of the current work is the restriction to anionic and amphiphilic legacy PFAA. It remains to be tested in each case if the current extraction procedure may be transferable to the wide range of modern PFAS. However, the recommended extraction with methanol and *EnvC* columns does not rely on the negative charge of the PFAA. It may also be useful for the extraction of PFAS with diverse properties, e.g., cations and zwitterions, which will be tested in the future. The application of the technique for the extraction of PFAA from human tissue samples yielded relatively clean extracts. The data did not indicate a particular accumulation of the PFAA in tissues included in the current study. The possible PFAA accumulation will be studied more thoroughly in an ongoing study comparing PFAA levels in tissue and plasma samples retrieved from the same individuals.

### Supplementary Information

Below is the link to the electronic supplementary material.

Supplementary file1 (DOCX 947 KB)

## Abbreviations

CV: Coefficient of variation
LC: Liquid chromatography
LOD: Limit of detection
LOQ: Limit of quantification
MS/MS: Tandem mass spectrometry
MTBE: Methyl-*tert*-butyl ether
PFAA: Perfluoroalkyl acids
PFAS: Per- and polyfluoroalkyl substances
PFBA: Perfluorobutanoic acid
PFBS: Perfluorobutane sulfonic acid
PFCA: Perfluoroalkyl carboxylic acids
PFDA: Perfluorodecanoic acid
PFDoA: Perfluorododecanoic acid
PFHpA: Perfluoroheptanoic acid
PFHpS: Perfluoroheptane sulfonic acid
PFHxA: Perfluorohexanoic acid
PFHxS: Perfluorohexane sulfonic acid
PFNA: Perfluorononanoic acid
PFOA: Perfluorooctanoic acid
PFOS: Perfluorooctane sulfonic acid
PFUdA: Perfluoroundecanoic acid
PFSA: Perfluoroalkyl sulfonic acids
S/N: Signal-to-noise ratio
SPE: Solid-phase extraction
TBA: Tetrabutylammonium
UPLC: Ultra-performance liquid chromatography
