# Supplementary material for: Less is more: a methodological assessment of extraction techniques for per- and polyfluoroalkyl substances (PFAS) analysis in mammalian tissues
Source: Anal Bioanal Chem. 2023 Aug 22;415(24):5925–38. doi: 10.1007/s00216-023-04867-5 (PMC10556126; doi:10.1007/s00216-023-04867-5)
Supplement: Supplementary file 1 — Supplementary file1 (DOCX 947 KB) [file 216_2023_4867_MOESM1_ESM.docx]

**Less is More: A Methodological Assessment of Extraction Techniques for Per- and Polyfluoroalkyl Substances (PFAS) Analysis in Mammalian Tissues**

Helena Mertens, Benedikt Noll, Tanja Schwerdtle, Klaus Abraham, Bernhard H. Monien

**Supplementary Information**

| **Table S1.** Parameter for the mass spectrometric detection of PFAAs and of the respective isotope-labeled standard compounds | | | | | | | | |
| --- | --- | --- | --- | --- | --- | --- | --- | --- |
| **analyte** | **RT** | **transition** | **Q1** | **Q3** | **DP** | **EP** | **CE** | **CXP** |
|  | (min) |  | (m/z) | (m/z) | (V) | (V) | (V) | (V) |
| **PFHxA** | 7.6 | quantifier | 313.1 | 268.9 | -5 | -10 | -14 | -23 |
|  |  | qualifier |  | 118.9 |  | -10 | -28 | -9 |
| **PFHpA** | 8.4 | quantifier | 363.1 | 318.9 | -5 | -10 | -14 | -9 |
|  |  | qualifier |  | 168.9 |  | -10 | -26 | -15 |
| **PFOA** | 8.9 | quantifier | 413.1 | 368.9 | -15 | -10 | -12 | -15 |
|  |  | qualifier |  | 168.8 |  | -10 | -24 | -15 |
| **PFNA** | 9.4 | quantifier | 463.1 | 419.0 | -10 | -10 | -14 | -11 |
|  |  | qualifier |  | 218.9 |  | -10 | -26 | -9 |
| **PFDA** | 9.8 | quantifier | 513.1 | 469.0 | -20 | -10 | -14 | -21 |
|  |  | qualifier |  | 218.9 |  | -10 | -24 | -15 |
| **PFUdA** | 10.1 | quantifier | 563.1 | 518.9 | -20 | -10 | -16 | -9 |
|  |  | qualifier |  | 268.9 |  | -10 | -22 | -17 |
| **PFDoA** | 10.4 | quantifier | 613.0 | 568.9 | -20 | -10 | -16 | -27 |
|  |  | qualifier |  | 268.9 |  | -10 | -30 | -9 |
| **PFTeDA** | 10.9 | quantifier | 713.1 | 668.9 | -25 | -10 | -18 | -29 |
|  |  | qualifier |  | 218.9 |  | -10 | -34 | -15 |
| **PFBS** | 6.8 | quantifier | 299.2 | 79.9 | -25 | -10 | -70 | -11 |
|  |  | qualifier |  | 98.9 |  | -10 | -38 | -9 |
| **PFHxS** | 8.4 | quantifier | 399.1 | 79.9 | -115 | -10 | -78 | -7 |
|  |  | qualifier |  | 98.9 |  | -10 | -76 | -11 |
| **PFOS** | 9.3 | quantifier | 499.1 | 98.9 | -105 | -10 | -122 | -15 |
|  |  | qualifier |  | 79.9 |  | -10 | -108 | -7 |
| **M5PFHxA** | 7.6 | quantifier | 317.9 | 272.9 | -5 | -10 | -14 | -23 |
| **M4PFHpA** | 8.4 | quantifier | 366.9 | 321.9 | -5 | -10 | -14 | -9 |
| **M8PFOA** | 8.9 | quantifier | 420.9 | 375.9 | -15 | -10 | -12 | -15 |
| **M9PFNA** | 9.4 | quantifier | 471.9 | 426.9 | -10 | -10 | -14 | -11 |
| **M6PFDA** | 9.8 | quantifier | 518.9 | 473.9 | -20 | -10 | -14 | -21 |
| **M7PFUdA** | 10.1 | quantifier | 569.9 | 524.9 | -20 | -10 | -16 | -9 |
| **MPFDoA** | 10.4 | quantifier | 614.9 | 569.9 | -20 | -10 | -16 | -27 |
| **M2PFTeDA** | 10.9 | quantifier | 714.9 | 669.9 | -25 | -10 | -18 | -29 |
| **M3PFBS** | 6.8 | quantifier | 301.9 | 79.9 | -25 | -10 | -70 | -11 |
| **M3PFHxS** | 8.4 | quantifier | 401.9 | 79.9 | -115 | -10 | -78 | -7 |
| **M8PFOS** | 9.3 | quantifier | 506.9 | 98.9 | -105 | -10 | -122 | -15 |

| 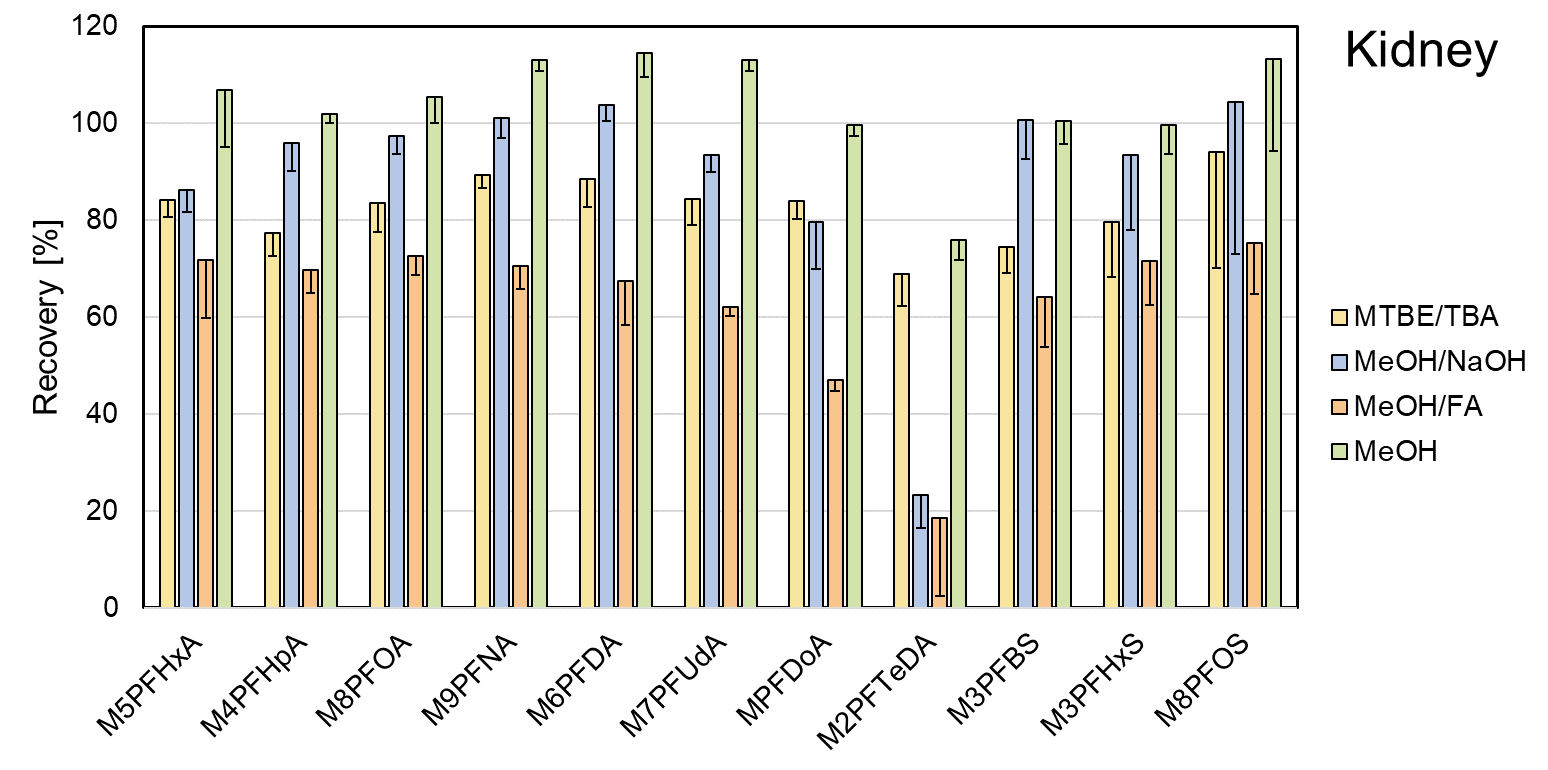 |
| --- |
| 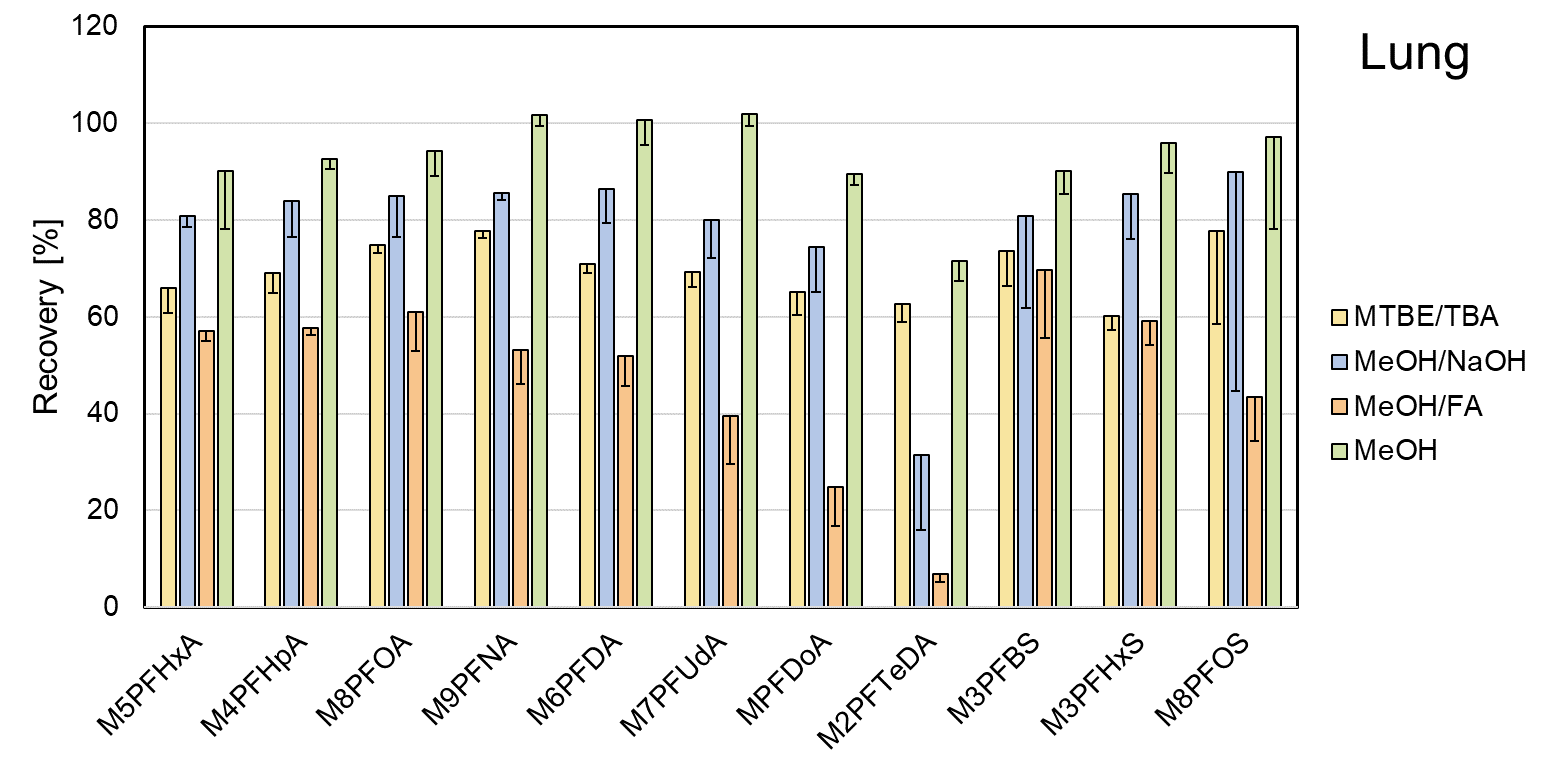 |
| **Fig. S1** Absolute recoveries (%) after fortification of kidney homogenates (upper panel) or lung homogenates (lower panel) with isotope-labeled PFAA (~ 190 ng/g) and extraction with MTBE after addition of TBA (yellow), 100 mM sodium hydroxide in methanol (blue), methanol/formic acid (1:1) (orange), or methanol alone (green). The bars and error bars show means and standard deviations of three samples. |

| 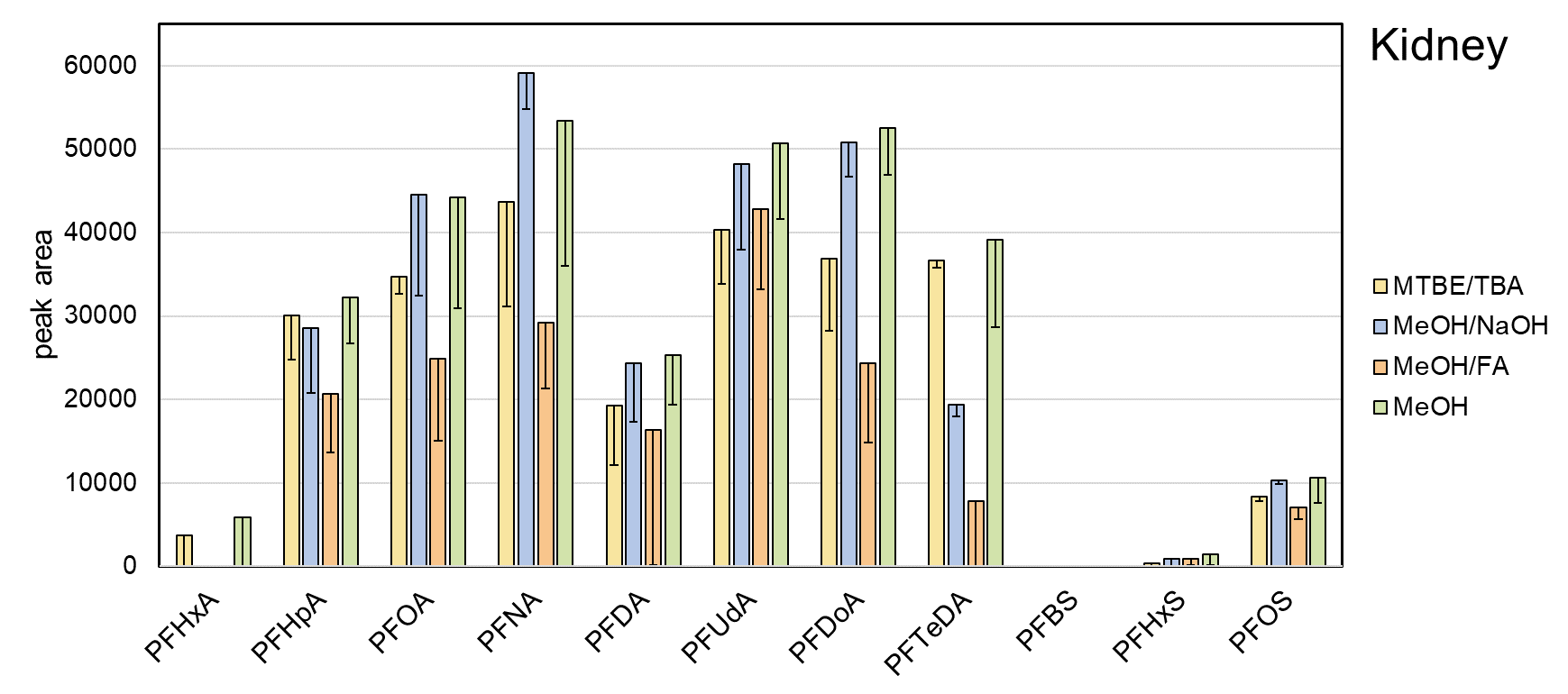 |
| --- |
| 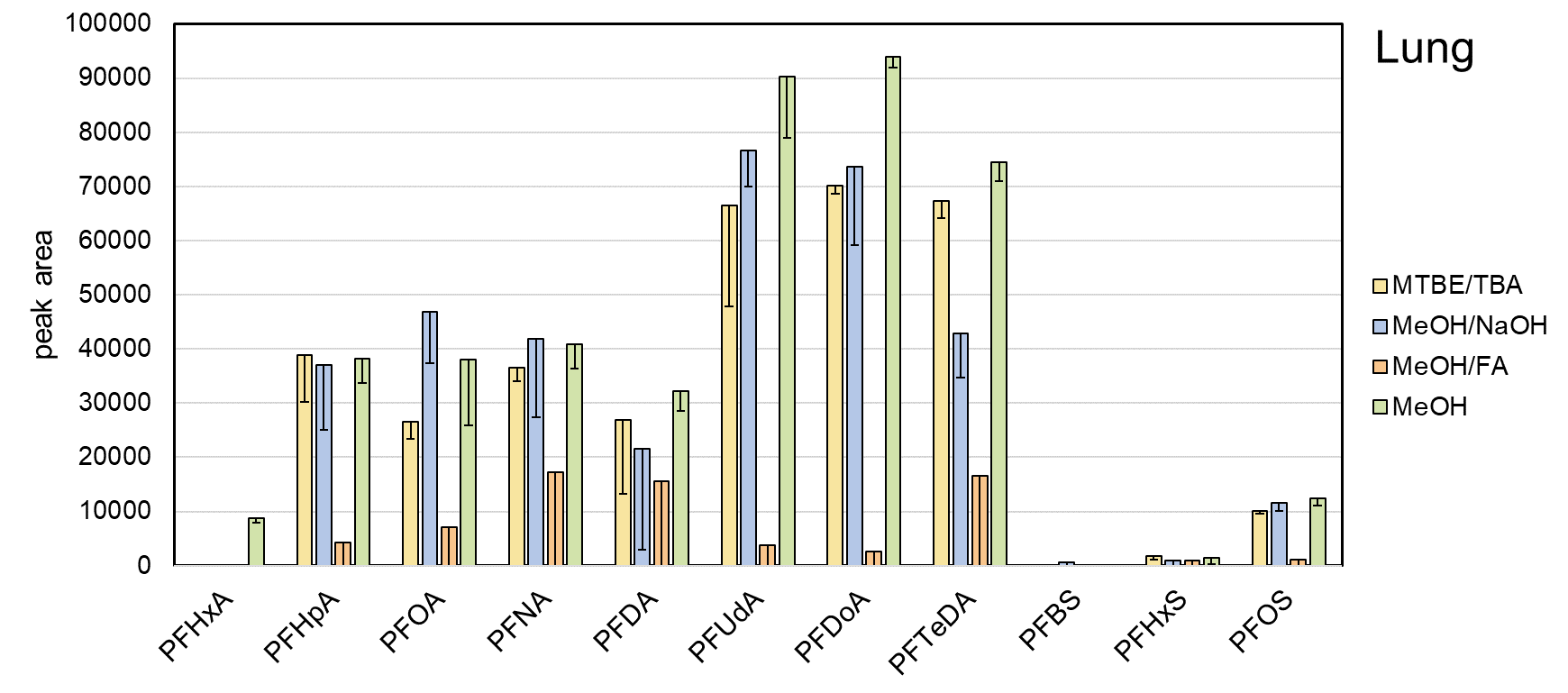 |
| **Fig. S2** Signal intensities reflect recoveries of PFAA extractions from kidney homogenates (upper panel) and lung homogenates (lower panel) with MTBE after addition of TBA (yellow), 100 mM sodium hydroxide in methanol (blue), methanol/formic acid (1:1) (orange), or methanol without any additives (green). The bars and error bars show means and standard deviations of three samples. |

| 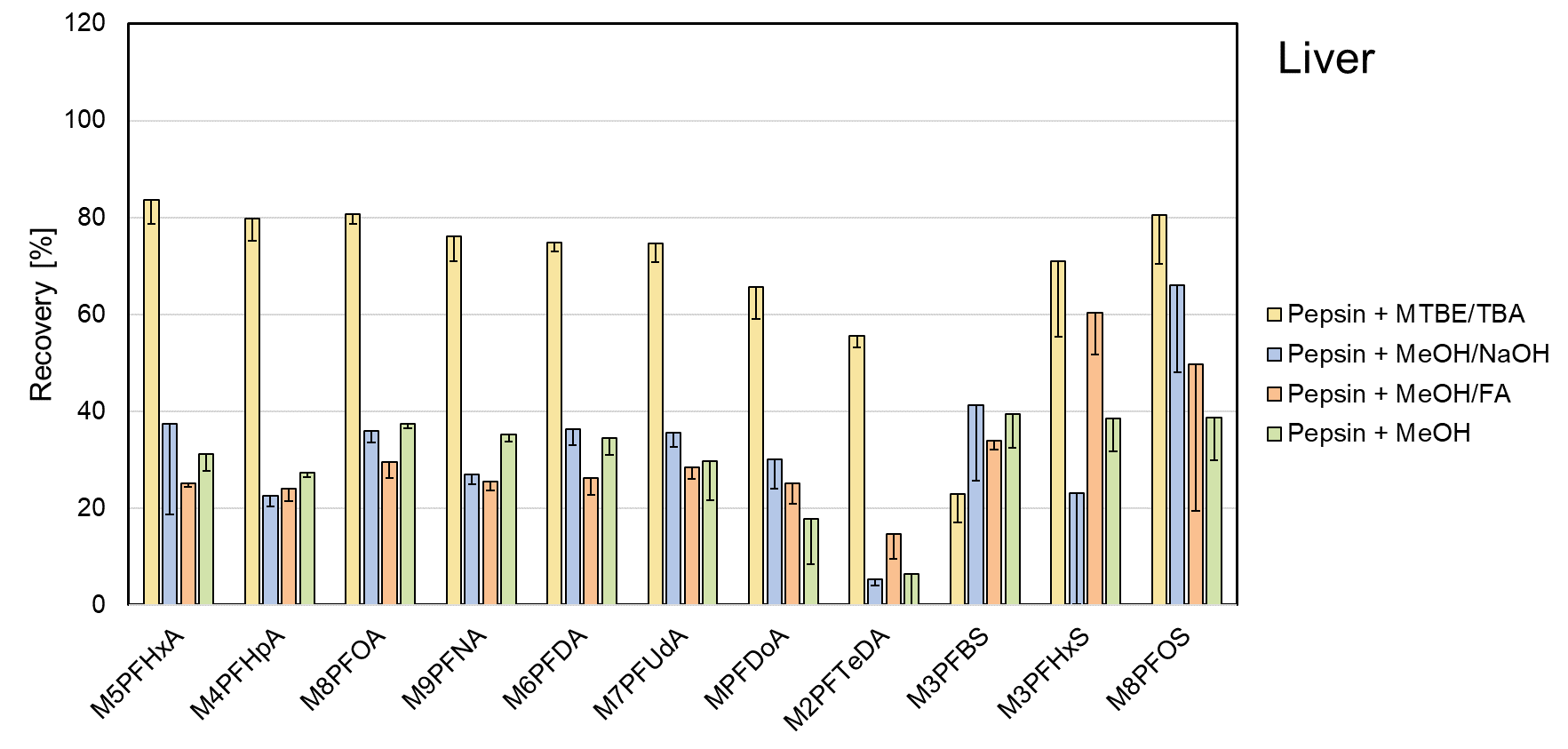 |
| --- |
| **Fig. S3** Absolute recoveries (%) after fortification of liver homogenates with isotope-labeled PFAA (~ 190 ng/g), pre-incubation of the samples with pepsin, and extraction with MTBE after addition of TBA (yellow), sodium hydroxide in methanol (blue), formic acid in methanol (orange), or methanol alone (green). The bars and error bars show means and standard deviations of three samples. The reduction of recoveries observed for the methanol-based extraction methods is probably due to the matrix enrichment in the extracts, which were visibly cloudier compared to those obtained without pepsin pre-incubation. |

| 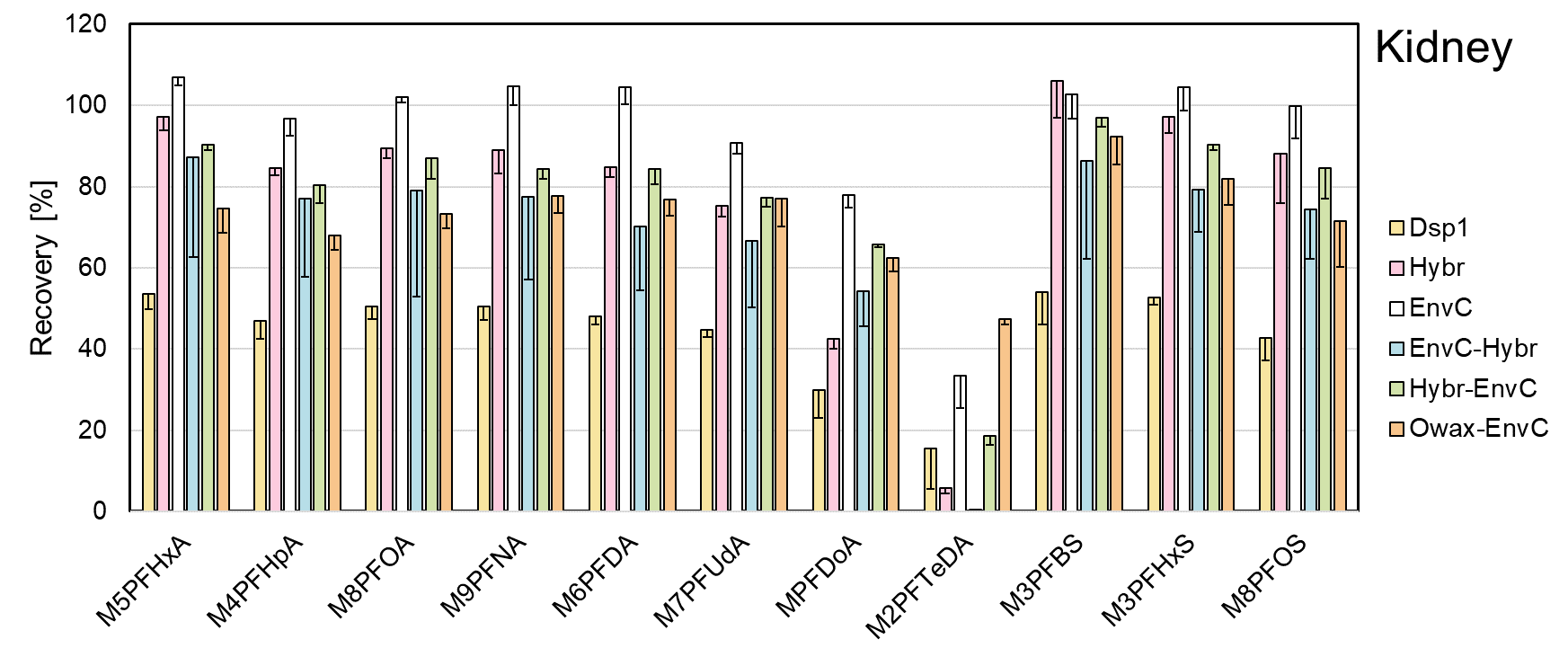 |
| --- |
| 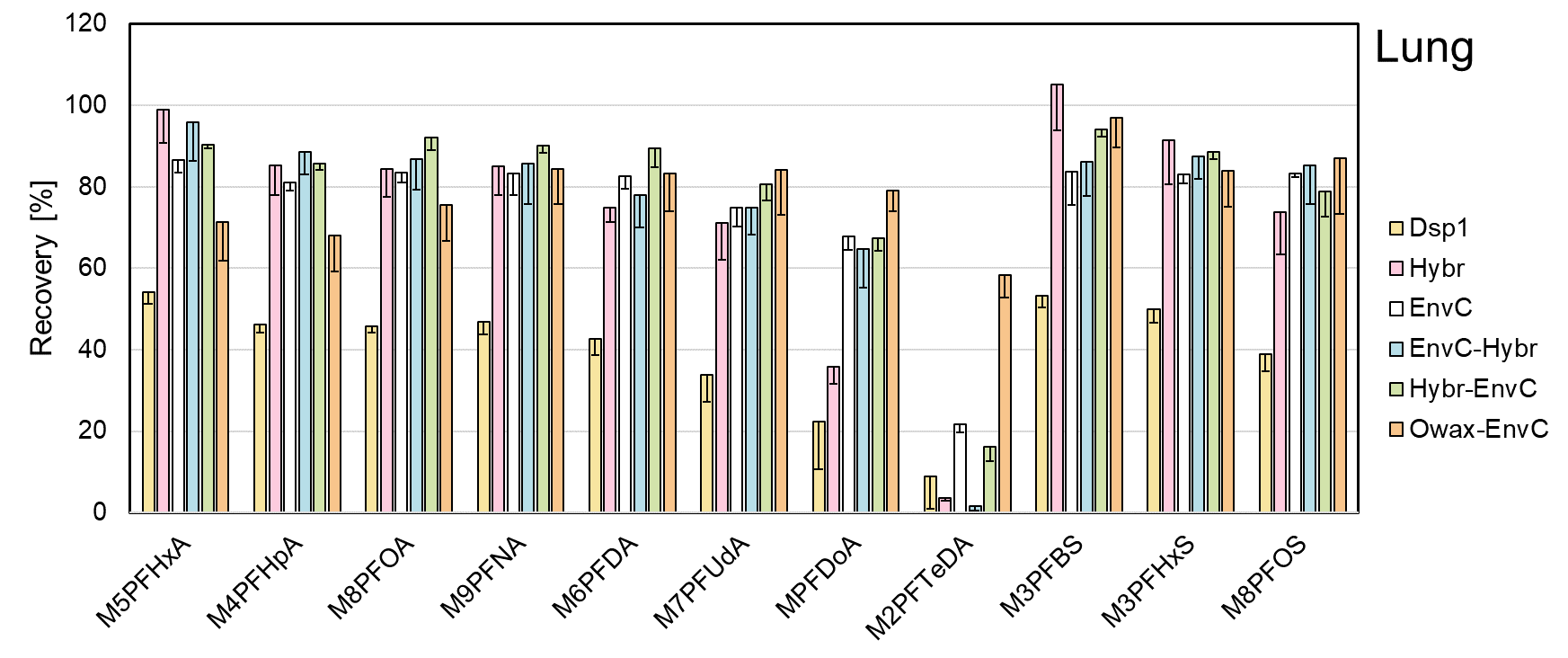 |
| **Fig. S4** Absolute recoveries (%) of isotope-labeled PFAAs from fortified wild boar kidney (upper panel) and lung (lower panel) after methanol extraction and application of different SPE methods using dispersive SPE *Dsp1* (yellow), the SPE columns *Hybr* (pink) or *EnvC* (white), or the combinations of the columns *EnvC-Hybr* (blue), *Hybr-EnvC* (green), or *OWax-EnvC* (orange). *Dsp2* was omitted for clarity and the *OWax* alone was not tested with homogenate extracts from lung and kidney. The bars and error bars show means and standard deviations of three samples. |

| 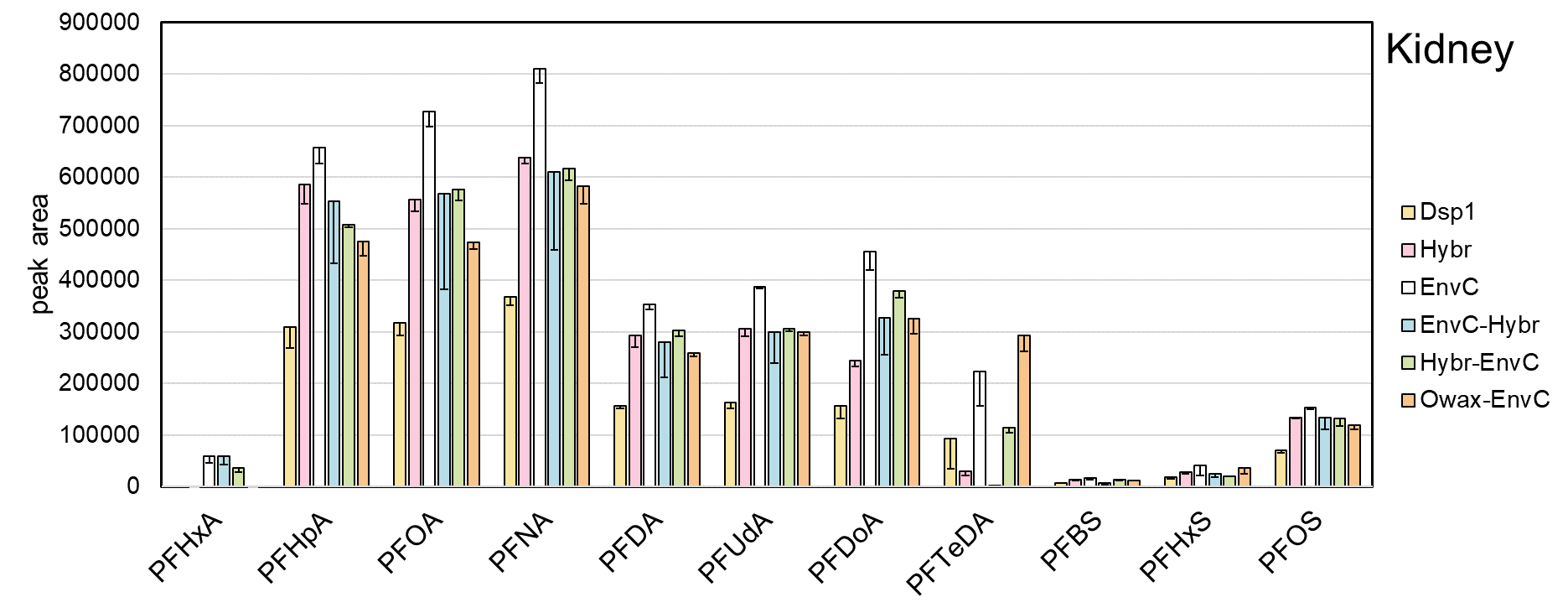 |
| --- |
| 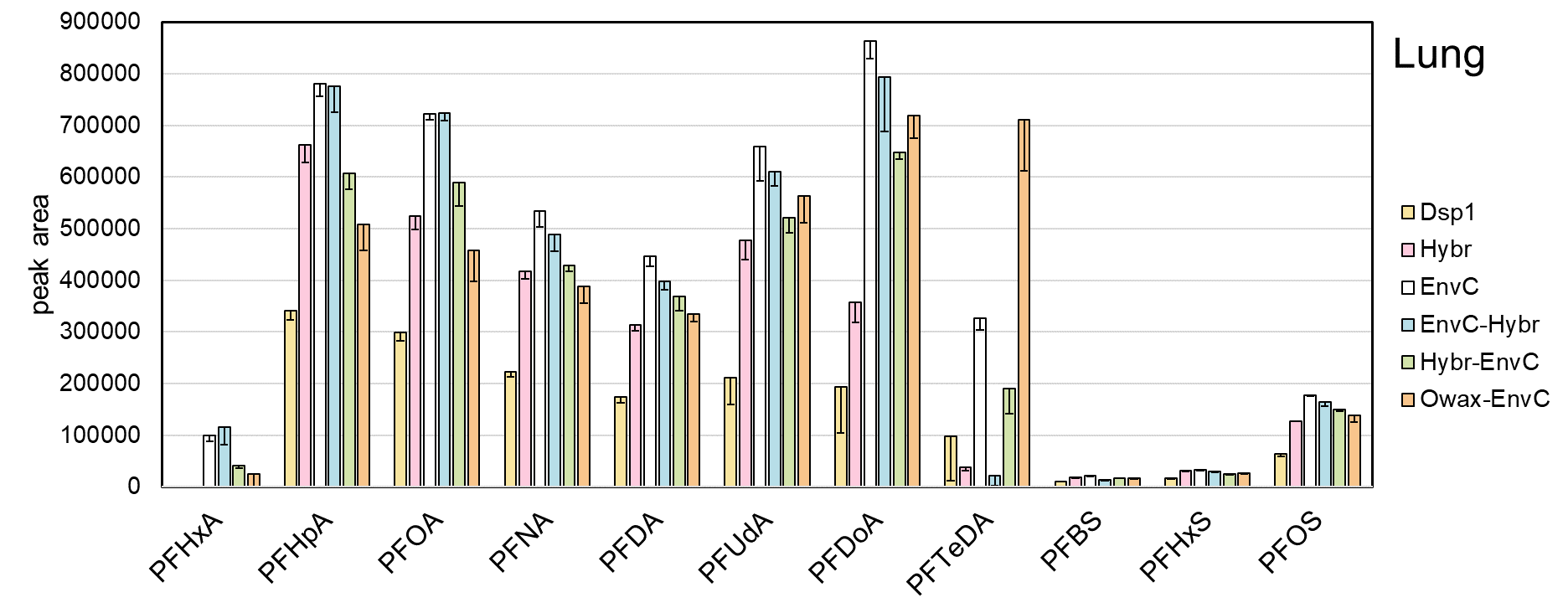 |
| **Fig. S5** Signal intensities of PFAA detection after methanol extraction and application of different SPE methods using dispersive SPE *Dsp1* (yellow), the SPE columns *Hybr* (pink) or *EnvC* (white), or the combinations of the columns *EnvC-Hybr* (blue), *Hybr-EnvC* (green), or *OWax-EnvC* (orange). *Dsp2* was omitted for clarity and the *OWax* alone was not tested with homogenate extracts from lung and kidney. The bars and error bars show means and standard deviations of three samples. |

| 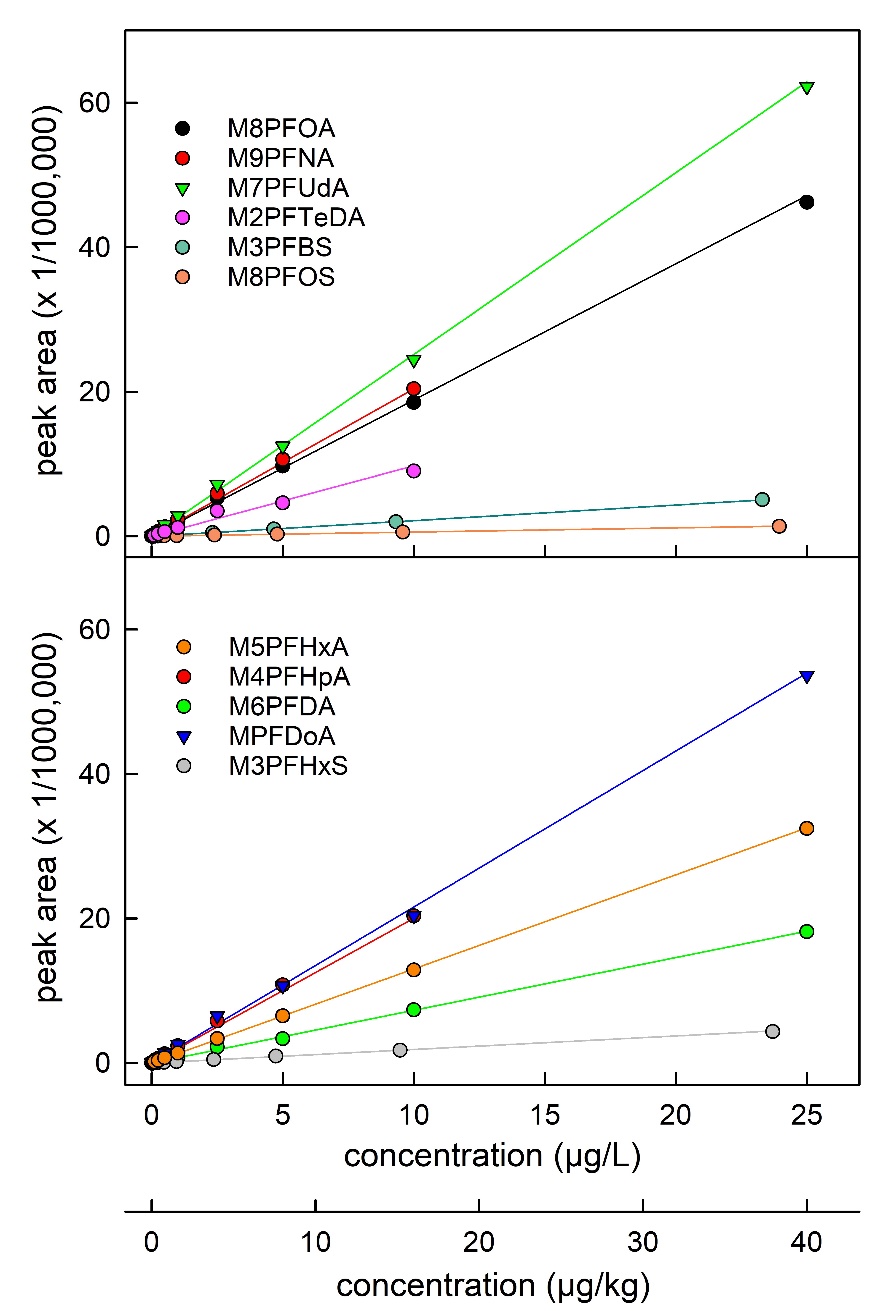 |
| --- |
| **Fig. S6** The linearity of detection was determined using 16 dilutions of the isotope-labeled standard mixture prepared from MPFAC-24ES (Wellington) with nominal concentrations between 0.00025 and 25 µg/L. The samples were injected in the presence of 125 mg processed wild boar liver homogenate. The concentrations of the standards solutions prepared from the alkali metal salts of M3PFBS, M3PFHxS and M8PFOS were corrected for the molecular weight of the respective ions. The data (distributed on two panels for enhanced clarity) were fitted with trend lines by linear regression with R in the range of 0.9870 and 0.9999. |


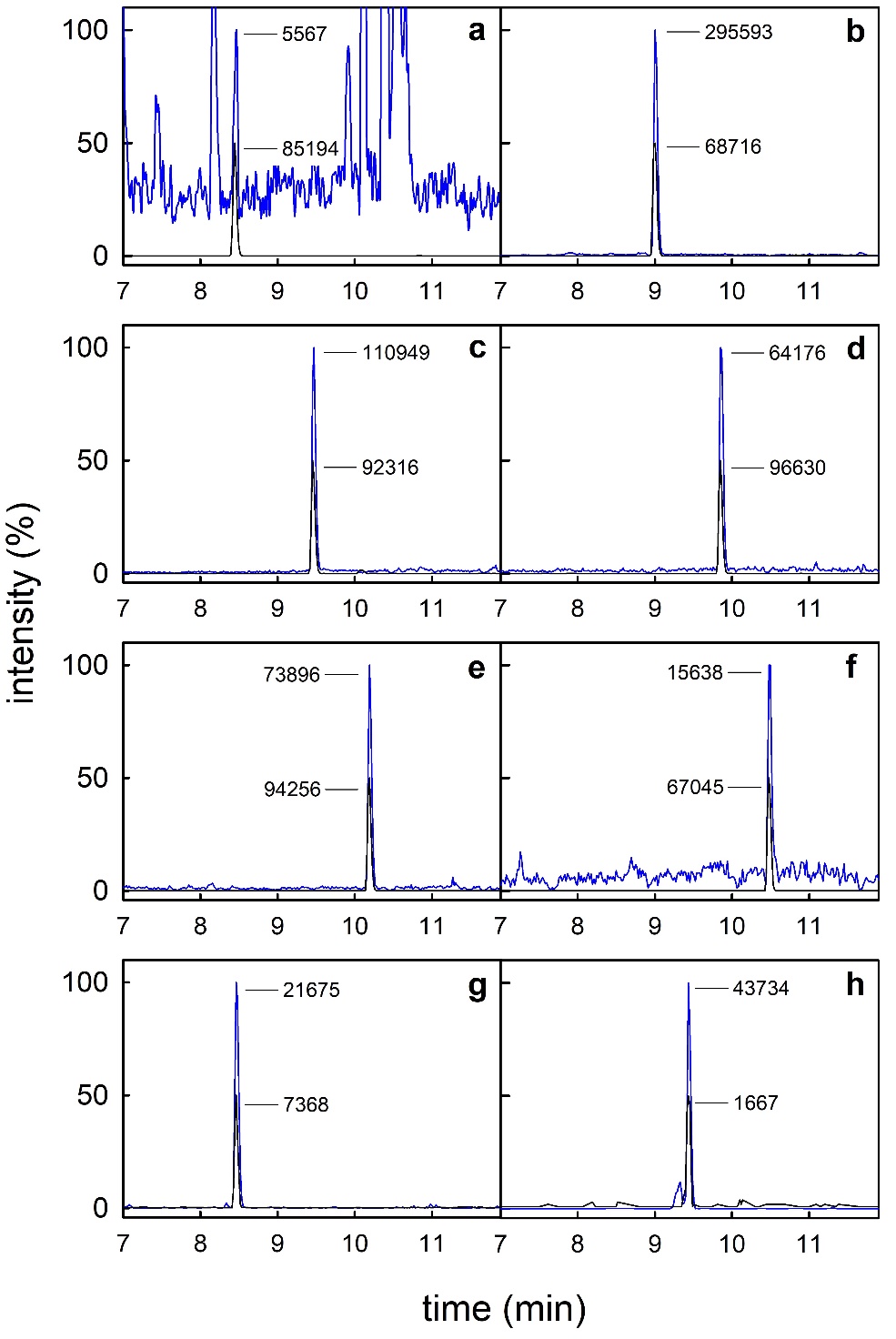


**Fig. S7** UPLC-MS/MS chromatograms of the quantifier traces (blue lines) from an extract sample of human lung containing PFHpA (a, *m/z* 363.1 → 318.9, < LOQ), PFOA (b, *m/z* 413.1 → 368.9), PFNA (c, *m/z* 463.1 → 419.0), PFDA (d, *m/z* 513.1 → 469.0), PFUdA (e, *m/z* 563.1 → 518.9), PFDoA (f, *m/z* 613.0 → 568.9), PFHxS (g, *m/z* 399.1 → 79.9), and PFOS (h, *m/z* 499.1 → 98.9), which were normalized to 100% signal intensity. The transitions of the respective isotope-labeled standard compounds (black lines) were adjusted to 50% signal intensity. Absolute intensities of analyte peaks and standard compounds are given.
